# LTBP-2 Has a Single High-Affinity Binding Site for FGF-2 and Blocks FGF-2-Induced Cell Proliferation

**DOI:** 10.1371/journal.pone.0135577

**Published:** 2015-08-11

**Authors:** Clementine Menz, Mahroo K. Parsi, Julian R. J. Adams, Mohamed A. Sideek, Zlatko Kopecki, Allison J. Cowin, Mark A. Gibson

**Affiliations:** 1 Discipline of Anatomy and Pathology, School of Medicine, University of Adelaide, Adelaide, South Australia, 5005, Australia; 2 Regenerative Medicine, Mawson Institute, University of South Australia, Adelaide, South Australia, 5095, Australia; Ottawa Hospital Research Institute, CANADA

## Abstract

Latent transforming growth factor-beta-1 binding protein-2 (LTBP-2) belongs to the fibrillin-LTBP superfamily of extracellular matrix proteins. LTBPs and fibrillins are involved in the sequestration and storage of latent growth factors, particularly transforming growth factor β (TGF-β), in tissues. Unlike other LTBPs, LTBP-2 does not covalently bind TGF-β, and its molecular functions remain unclear. We are screening LTBP-2 for binding to other growth factors and have found very strong saturable binding to fibroblast growth factor-2 (FGF-2) (Kd = 1.1 nM). Using a series of recombinant LTBP-2 fragments a single binding site for FGF-2 was identified in a central region of LTBP-2 consisting of six tandem epidermal growth factor-like (EGF-like) motifs (EGFs 9–14). This region was also shown to contain a heparin/heparan sulphate-binding site. FGF-2 stimulation of fibroblast proliferation was completely negated by the addition of 5-fold molar excess of LTBP-2 to the assay. Confocal microscopy showed strong co-localisation of LTBP-2 and FGF-2 in fibrotic keloid tissue suggesting that the two proteins may interact in vivo. Overall the study indicates that LTBP-2 is a potent inhibitor of FGF-2 that may influence FGF-2 bioactivity during wound repair particularly in fibrotic tissues.

## Introduction

Latent transforming growth factor-beta-1 binding protein-2 (LTBP-2) is a member of the fibrillin-LTBP superfamily of extracellular matrix proteins. These proteins are all structurally similar, consisting of a rod-like molecule of tandem EGF-like 6-cys repeats interspersed with characteristic 8-cys motifs [1–5]. Fibrillins 1–3 form microfibrils which, together with a core of elastin, are the main structural components of elastic fibers [2, 5]. LTBPs -1, 3, and 4, covalently bind latent growth factor TGF-β and direct the growth factor to storage depots within the extracellular matrix [1, 6]. Fibrillin microfibrils are considered to be a principal storage location for these latent complexes and they act as critical regulators of TGF-β activation [7].

Structurally, LTBP-2 is more similar to the other LTBPs than fibrillins, but like fibrillins, it does not directly bind TGF-β [8, 9] and LTBP-2 function remains largely unclear. An early study reporting that LTBP-2 null mice have embryonic lethality [10], has recently been contradicted by Inoue et al. who presented a LTBP-2 null mouse with only a mild ocular phenotype [11]. This result agrees more closely with LTBP-2 null humans who also have mild ocular phenotypes including glaucoma, megalocornea, ectopis lentis and microspherophakia [12–15]. It has long been documented that LTBP-2 is associated with elastic fibers in developing elastic tissues [8] and there is evidence that LTBP-2 may play a negative regulatory role in elastinogenesis, inhibiting tropoelastin interactions with fibulin-5 and heparan sulphate proteoglycans [16]. In vitro studies have shown that LTBP-2 specifically binds to fibrillin-1 rather than fibrillin-2 and that LTBP-2 can compete with LTBP-1 for binding to the fibrillin-1 molecule, suggesting that LTBP-2 may indirectly affect TGF-β bioavailability [17]. This idea is supported by a recent study linking LTBP-2 gene mutations to a recessive form of Weill—Marchesani syndrome (WMS) [18] which is characterized by short stature, brachydactyly, thick fibrotic skin and ectopia lentis (WMS, Online Mendelian Inheritance in Man # 608328). This finding clearly links LTBP-2 to fibrillin biology as mutations in the fibrillin-1 gene also cause some presentations of WMS [19]. Fibrillin-1 gene mutations also cause Marfan Syndrome (MFS) (OMIM number 154700) and many of the characteristics of WMS and MFS have been attributed to aberrant TGF-β signaling [20]. However fibrillins and associated MAGP proteins have been documented to bind many other growth factors in latent and/or active forms, including bone morphogenic proteins (BMPs) 2, 4, 5, 7 and 10, and connective tissue growth factor [21–24]. Thus sequestration or release of these molecules may also influence microfibril modulation of growth factor signaling and contribute to aberrant microfibril function in these genetic disorders and other diseases.

Given the above evidence it seems clear that LTBP-2 also has some as yet unidentified role in modulation of growth factor storage and activity. To investigate we have commenced screening LTBP-2 with candidate growth factor binding partners. In this paper we report a very strong interaction of LTBP-2 with fibroblast growth factor-2 (FGF-2). FGF-2 or basic FGF is an important member of a family of cytokines now numbering over 20, that modulate cellular behaviour through activation of FGF receptors (FGFRs)[25]. FGF-2 promotes proliferation, differentiation and migration in fibroblasts and a variety of other cell types [26] and has influence on a range of processes including angiogenesis, tissue remodeling, wound healing and tumour growth [27–29]. FGF-2 has prominent roles in the repair and regeneration stages of wound repair. In acute wound healing, FGF-2 promotes tissue repair by stimulating fibroblast motility and collagenase production for extracellular matrix remodeling, promoting granulation tissue formation, and increasing keratinocyte motility during re-epithelialization [30]. In chronic wounds such as hypertrophic scars and keloids, the growth factor can attenuate fibrosis and promote healing by down-regulating TGF-β induced collagen production, increasing matrix degrading enzymes such as matrix metalloprotein-1 and inducing myofibroblast apoptosis [31]. A role for FGF-2 in microfibril biology has yet to be documented.

We have found that FGF-2 has a single high-affinity binding site in a central region of LTBP-2. In addition LTBP-2 inhibited FGF-2 induced fibroblast proliferation in a bioassay and confocal microscopy showed strong co-localisation of LTBP-2 and FGF-2 in fibrotic keloid skin.

## Materials and Methods

Rabbit anti-[human LTBP-2 peptide] antibody 3504 has been described previously [17]. Anti-His_4_ antibodies were purchased from Qiagen (Valencia, CA). FGF-2 antibody (#610871) for immunohistochemistry was supplied by BD labs. Recombinant human FGF-2, VEGF, BMP-4, and BMP-7 and corresponding antibody detection systems (duo-set kits) were obtained from R and D systems. Mouse anti-fibrillin-1 monoclonal antibody MAB1919 and rabbit anti-phospho-FGFR1(Tyr653/Tyr654) antibody were obtained from Merck Millipore, Germany. Rabbit monoclonal anti-total FGFR1 antibody (D8E4) was purchased from Cell Signalling Technology (Danvers, MA).

### Recombinant protein production and purification

**Fig 1:**
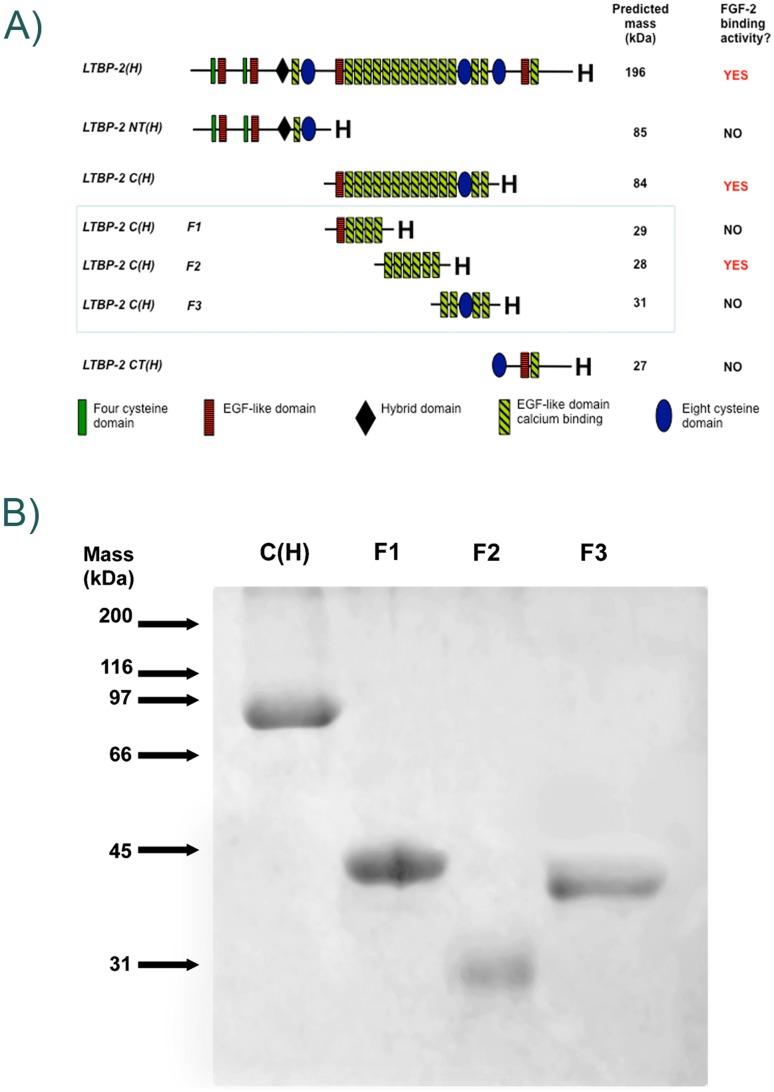
Recombinant LTBP-2 Fragments. **A**. Schematic diagram of recombinant LTBP-2 fragments. Protein fragments generated specifically for this study (LTBP-2C(H) F1, F2 and F3) are highlighted within the blue box. FGF-2 binding was confined to a single central region of the LTBP-2 molecule consisting of 6 EGF-like repeats (fragment LTBP-2C(H) F2).**B**. SDS-PAGE of purified recombinant LTBP-2 fragments. Samples of purified fragments LTBP-2 C(H), LTBP-2 C(H) F1, LTBP-2 C(H) F2 and LTBP-2 C(H) F3 were analyzed on a 12% gel under non-reducing conditions and stained with Coomassie blue. The relative mobilities of protein standards are indicated by arrows.

CDNAs encoding recombinant human LTBP-2 and contiguous fragments NT(H), C(H) and CT(H) were cloned into episomal expression vector pCEP4 as described previously [32]. In addition, three contiguous sub-fragments of LTBP-2C(H), entitled F1, F2 and F3, were generated for the current study (Fig 1). Briefly, cDNAs encoding these sub-fragments were amplified by PCR from the LTBP-2C (H) cDNA [32] and ligated into a modified episomal expression vector BM40:his_6_:pCEP-4, such that each recombinant fragment was flanked by a BM-40 secretion-signal peptide and a C-terminal His_6_ tag. PCR amplification with Pfu turbo Cx DNA polymerase (Stratagene) used 25 cycles of 95°C for 30 sec, annealing for 30 sec and extension for 1 min at 72°C. For LTBP-2 C(H) F1, cDNA bases 775 to 1581 were amplified using sense primer 5′-CTGAAAGCTTGGACTCTCAGGCTGGCCAGG-3′ and antisense primer 5’CACAAAGCTTCACATCTTGGCAGCCCTTCTCAT- 3’, with an annealing temperature of 54°C, giving a product of 807 bp. For LTBP-2 C(H) F2, cDNA bases 1553 to 2343 were amplified using sense primer 5’- GAAGGGCTGCCAAAGCTTGGATGAGTGTGCCAG- 3’ and antisense primer 5’- CGTTAAGCTTCTCGTCTATGTCAATGCAG- 3’, with an annealing temperature of 62°C, giving a product of 791bp. For LTBP-2 C(H) F3, cDNA bases 2315 to 3180 were amplified using sense primer 5’-CTCCATTGAAAGCTTCGAGTGCGCCAACGACAC- 3’ and antisense primer 5′-TTTTAAGCTTGATGTCCATGTGGATGTCGT-3′, with an annealing temperature of 54°C, giving a product of 866 bp. The PCR products were purified by excision from agarose gels, A-tailed and ligated into the pGEM-T easy plasmid, as described previously [17]. The ligated constructs were transformed into JM109 competent cells, and individual clones were propagated and sequenced. Error-free cDNAs were then excised by digestion with HindIII, and ligated into the HindIII site of the modified pCEP4 vector [17]. These expression constructs were transfected into 293-EBNA cells, and cells were grown with selection antibiotic hygromycin B. The cell culture medium was harvested and recombinant protein was purified using chelating Ni-Sepharose as previously described [33]. Samples of the recombinant proteins were dialyzed into TBS-0.5M NaCl, and analyzed by SDS-PAGE on a 12% gel under both reducing and non-reducing conditions to confirm size and purity.

### Solid Phase Binding Assays

Solid phase binding assays were conducted as described previously [34] using an adaptation of the method provided with the growth factor DuoSet ELISA Development kits (R&D Systems). Briefly recombinant LTBP-2 or fragments was coated overnight onto microtitre wells, with BSA-coated wells as negative controls. After washing, growth factor, usually FGF-2, was added to the wells, at either a constant concentration, or in increasing amounts for the saturation curve, and incubation was continued for 2 h. After thorough washing, bound growth factor was detected by incubation for 2 h with biotinylated detection antibody (2.5 μg/ml) followed by 20 min with streptavidin conjugated with horseradish peroxidase. Colour development (absorbance at 450 nm) was detected using TMB substrate and quenched using 1M H_2_SO_4_. For the FGF-2 saturation binding curves, the amount of FGF-2 bound was calculated from a concurrent standard ELISA for FGF-2, following the protocol provided in the DuoSet ELISA Development kit. The Kd for each interaction was calculated by non-linear regression analysis of this saturation curve using GraphPad Prism (Version 4).

For heparin binding studies, binding assays were conducted with heparin-albumin conjugate [HAC] as described previously [32]. Briefly wells were coated overnight with HAC (or BSA control) in TBS, followed by blocking with 5% milk-TBS for 1 hour. LTBP-2C (H) or fragments F1, F2 and F3 were added to wells in TBS+2mM CaCl_2_, and incubated at 37°C for 3 hours. Following washing, primary antibody (usually anti-pentaHis) in TBS+2mM CaCl_2_ was added to wells, and incubated at 37°C for 2.5 h. Binding was detected using goat anti[mouse IgG] antibody-HRP conjugate followed by substrate development as described above.

### Cell proliferation assay

Human foreskin fibroblasts (passage 4) were suspended in DMEM plus 10% fetal calf serum, non-essential amino acids, and penicillin/streptomycin. The cells were plated at a density of 4 x 10^4^ cells per well into a 96-well culture plate (Costar #3596, Corning) and incubated overnight at 37°C in a cell culture incubator (5% CO_2_). The wells were rinsed with serum-free DMEM and further incubated at 37°C for 48 h in 100 μl of serum-free DMEM containing FGF-2 (10 ng/ ml, 0.625 nM). For some wells, the FGF-2 was pre-incubated for 15 min with five or ten fold molar excess of LTBP-2 or fragment LTBP-2C F2 prior to addition of the mixture to the wells. Follistatin (30 ng/well) was included in some incubations to ensure that observed effects were not due to undetectable quantities of TGF-β in the recombinant LTBP-2 preparations [35]. Cell proliferation was measured using metabolic substrate WST-1 (Roche) following the manufacturer's instructions. Briefly 10 μl of WST-1 was added to each well. The plate was rocked gently for 1 min to mix then returned to the cell culture incubator for 30 mins. A microplate spectophotometer (Shimadzu UV-1601) was then used to read absorbances at 450 nm, and 595 nm and the reading at 595 nm was subtracted from the 450 nm reading to give final colour values.

### Detection and quantitation of FGFR1 activation

Human foreskin fibroblasts were plated at a density of 4 x 10^5^ cells per well into a 6-well plate (Nunclon Surface, Nalge Nunc International, Denmark) and incubated overnight at 37°C in a cell culture incubator (5% CO_2_) in DMEM plus 10% fetal calf serum. The wells were rinsed with PBS and further incubated at 37°C for 2 hours with 1 ml of serum-free DMEM containing FGF-2 (10ng/ml, 0.625 nM). For some wells, the FGF-2 was preincubated for 15 min with 10-fold molar excess of LTBP-2 or fragment LTBP-2C F2 prior to addition to the wells. The cells were then lysed using extraction buffer (containing 50 mM Tris (pH 6.8), 0.5% SDS, 2 mM EDTA and cocktails of phosphatase and protease inhibitors [#04906837001 and #11836153001, Roche, Germany]) and analysed by SDS-PAGE on 12% gels (100 ug cell protein per well). The proteins were immunoblotted onto nitrocellulose membranes (0.2 μm, Pall Corporation, Pensacola, FL) as described previously [34]. The membranes were blocked in a 5% skim milk in TBS-Tween20 for 1 hr at RT with gentle shaking. The blots were horizontally cut into 2 halves and incubated at 4°C overnight with 0.2ug/ml of anti-phospho-FGFR-1(Tyr653/Tyr654) antibody (#06–1433, Millipore, CA) or anti-total FGFR1 antibody (#9740, CST, MA) (upper half) or 1ug/ml of anti-β actin antibody (#SANTSC-47778, Santa Cruz Biotechnology, Inc, USA) (lower half). After washing the blot halves with TBS-Tween, bands were visualised with the appropriate anti-rabbit IgG or anti-mouse IgG antibodies conjugated with IR800 fluorescence dye (#SA5-35571 or #SA5-35521, Thermo Scientific, U.S.A). Membranes were imaged with the LI-COR Odyssey Infrared Imaging System. Bands were quantitated using ImageJ 1.48 software [National Institutes of Health (NIH), Bethesda, MD] and normalised to the internal β-actin signal. For comparison of the phospho-FGFR1 signal between samples, the ratio of the normalised phospho-FGFR1 signal to the total FGR1 signal was expressed as a percentage relative to the average value from cells treated with FGF-2 only (equaling 100%).

### Immunohistochemistry

Paraffin-embedded tissue blocks of tissue from normal skin and keloid were prepared from biopsy or discarded surgery material from adult human subjects with informed written consent which is archived and human ethics clearances from the University of Adelaide (#H-16-2001) and the Calvery Hospital Research Ethics Committee (11-CHREC-F007). Sections (4 μm thickness) were cut via a microtome and dewaxed in xylene for 30 min and rehydrated gradually for 2 min each through a series of ethanol solutions (100% to 30%) followed by water and finally PBS. The slides were then placed in 15% target retrieval solution [36] for 60 mins, starting at 90°C and dropping to 65°C. The sections were washed in PBS, incubated with trypsin (0.025% w/v) for 3 min at 37°C then blocked with 3% normal goat serum for 30 min. After washing with PBS, the sections were incubated overnight at 4°C with primary antibodies (2 or 2.5 μg/ml) or matched concentrations of appropriate rabbit or mouse IgG as negative controls. After thorough washing in PBS, the sections were incubated for 1 h with a 1:200 dilution of appropriate secondary antibody (anti-rabbit IgG antibody conjugated to fluor Alexa 488 or anti-mouse IgG antibody conjugated to Alexa 594, Life Technologies). After further washing with PBS the sections were treated with 0.1μg / ml of 4′,6-Diamidino-2-phenylindole dihydrochloride (DAPI) [Sigma; D9542] and sealed under a coverslip in Dako fluorescence mounting medium. The slides were examined using a Leica TCS SP5 confocal microscope, sequentially excited at 488 nm for Alexa 488 (emission window 496–533 nm), 561 nm for Alexa 594 (emission window 569–753 nm) and 405 nm for DAPI (emission window 413–460 nm). For quantitation, 3 random areas (each 0.038 mm^2^) per section were analysed using the AnalySIS software package (Soft-Imaging System, Munster, Germany).

## Results and Discussion

### FGF-2 has a strong affinity for LTBP-2

Expression constructs in a modified pCEP4 vector for full-length human LTBP-2 and three contiguous fragments spanning the molecule have been described previously [32]. In addition similar constructs encoding three smaller recombinant fragments spanning the central region of the LTBP-2 molecule were made, each encoding an N-terminal BM40 signal peptide and a C-terminal His6 tag (Fig 1A). Each encoded fragment was produced in 293-EBNA cells and purified from the culture medium as previously described [32]. Each protein fragment gave a single band on SDS-PAGE (Fig 1B) indicating a high degree of purity. Fragments LTBP-2C F1, F2 and F3 (predicted molecule weights of 29 kDa, 28 kDa and 31 kDa respectively) migrated under non-reducing conditions with apparent molecular weights of 40 kDa, 30 kDa and 37 kDa respectively.

**Fig 2:**
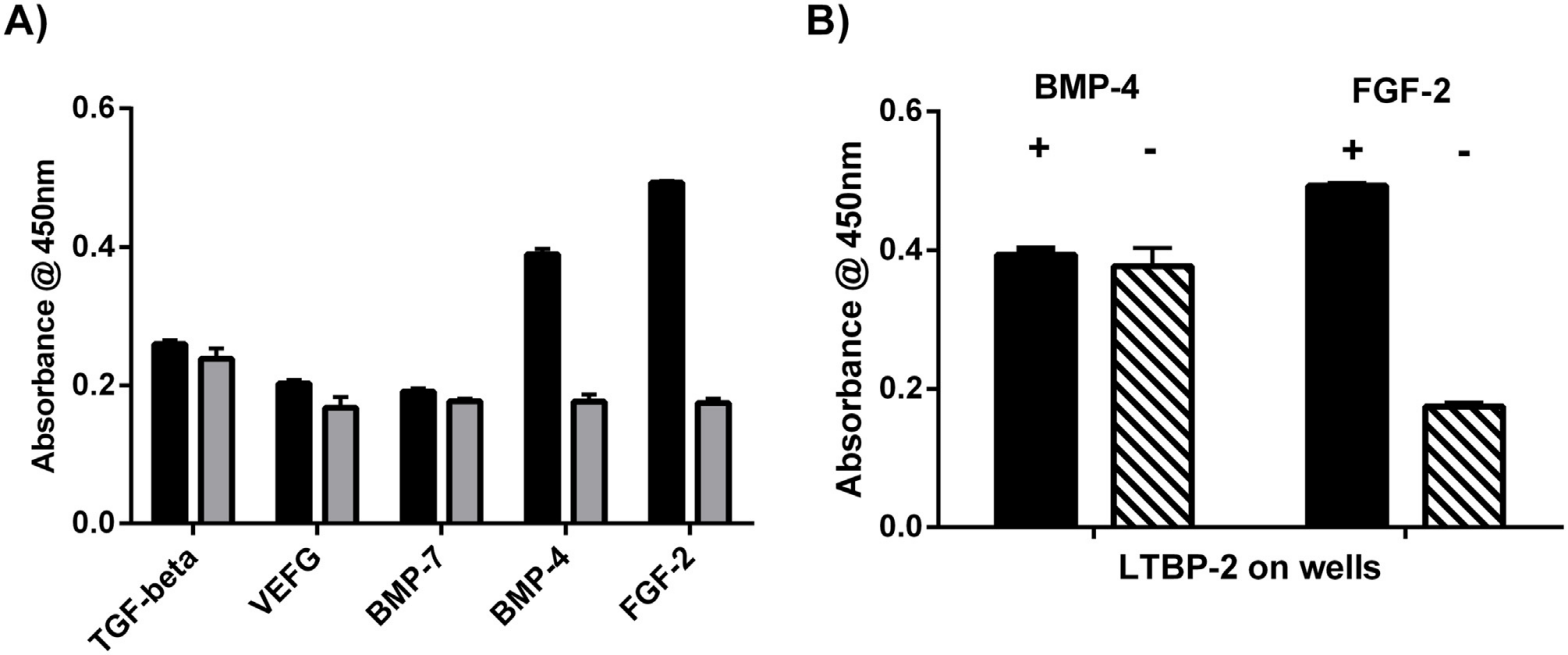
LTBP-2 specifically binds FGF-2 but not VEGF, BMP-4, BMP-7 or TGF-beta. **A**. Microtitre wells were coated with rLTBP-2 (black columns) or BSA(shaded columns) (100 ng/ well). After blocking, triplicate wells were incubated at 37°C for 2h with TGF-beta (13 ng / well), VEGF (21 ng / well), BMP-7 (4 ng/well), BMP-4 (4 ng / well) or FGF-2 (10 ng / well). Growth factor binding was detected using specific biotinylated antibodies from Duoset kits as described in material and methods. Mean values ± S.D. from triplicate wells are shown. **B**. Microtitre wells were coated with rLTBP-2 (100ng/well) was coated onto microtitre plates. After blocking, triplicate wells were incubated at 37°C for 2h with (black columns) or without (cross-hatched) growth factor, (BMP-4 (4ng/well) or FGF-2 (10ng/well). Binding of growth factor to LTBP-2 was detected using biotinylated anti-BMP-4 detection antibody (0.5ug/ml) or anti-FGF-2 detection antibody (0.25ug/ml), followed by a peroxidase detection method (see material and methods). Mean values ± S.D. from triplicate wells are shown. Note the anti-BMP-4 antibody bound to the wells equally strongly in the presence or absence of added BMP-4, indicating the interaction was non-specific.

**Fig 3:**
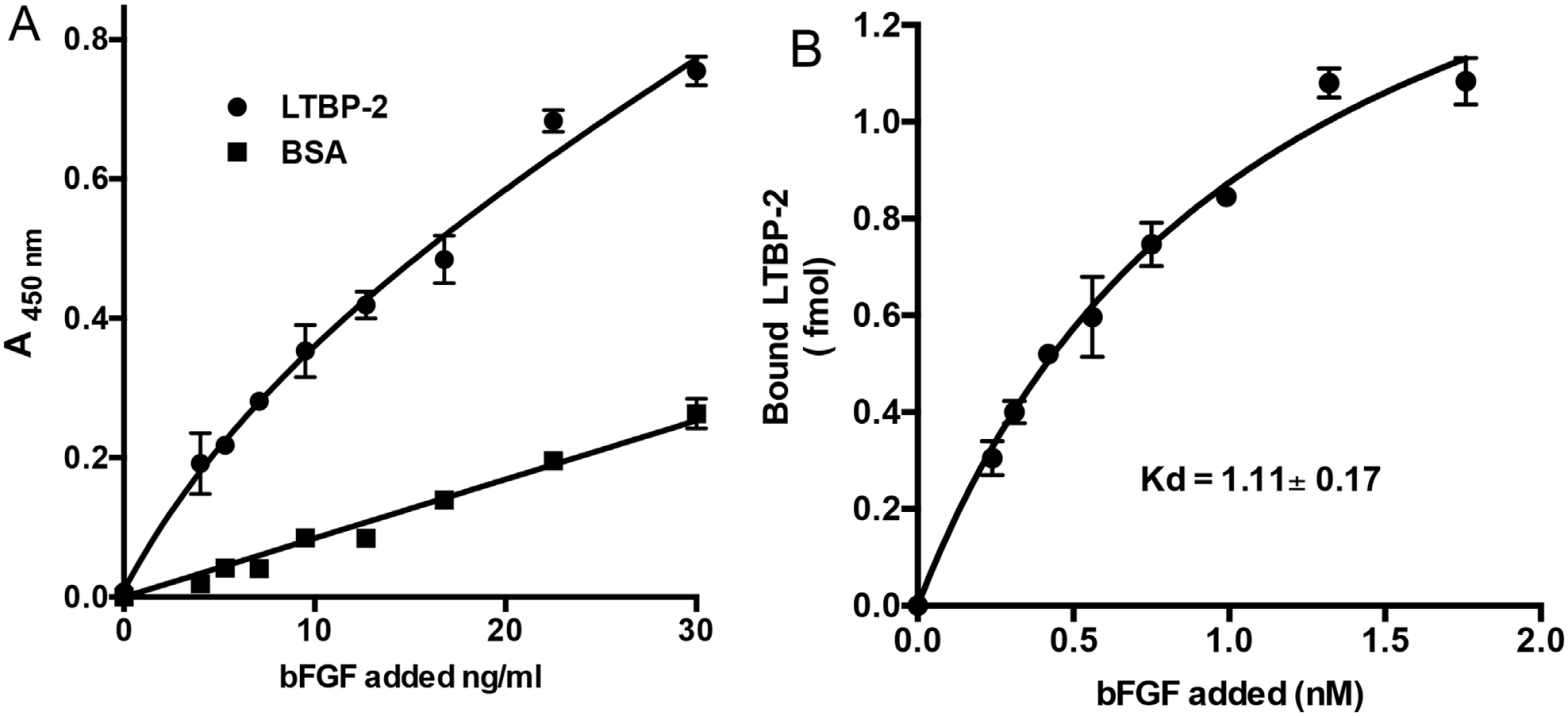
LTBP-2 interacts strongly with FGF-2. **A**. Microtitre wells were coated with 200 ng rLTBP-2 or BSA control. After blocking, triplicate wells were incubated with 0–1.8 nM concentrations of FGF-2 (0–30 ng/ml) for 3 h at 37°C. FGF-2 binding was detected following sequential incubation of the wells with biotinylated mouse anti-[human FGF-2] antibody and streptavidin-HRP conjugate following the duoset protocol. Circles, LTBP-2; squares, BSA. Mean values ± S.D. of triplicate determinations are shown. **B**. Kd calculation. Following subtraction of the average BSA signal, the A450nm values were converted to fmol of FGF-2 using a standard ELISA curve (not shown). An additional graph was plotted of bound versus added FGF-2 and the Kd for interaction with LTBP-2 was calculated by non-linear regression analysis of the curve using the prism 4.0 program. Mean values ± S.D. from triplicate determinations are shown.

Full-length recombinant LTBP-2 was tested for binding to a range of growth factors including vascular endothelial growth factor, BMP-4, BMP-7 and FGF-2 in an established solid phase binding assay (Fig 2A) [34]. Initial screening identified FGF-2 and BMP-4 as candidate binding partners for LTBP-2. However a further experiment identified BMP-4 as a false positive, as the BMP-4 antibody showed binding to the LTBP-2 coated wells in the absence of BMP-4 protein (Fig 2B). Of the growth factors tested only FGF-2 showed strong saturable binding to LTBP-2 (Fig 3A). The binding curve was quantitated from a standard ELISA curve for FGF-2 coated onto microtitre wells. This enabled the Kd for the LTBP-2 / FGF-2 interaction to be calculated by non-linear regression analysis of the curve produced by plotting amount FGF-2 bound versus concentration of FGF-2 added (Fig 3B). The prism program calculated the Kd as 1.11 ± 0.17 nM for a single binding site. This finding indicated that the LTBP-2 / FGF-2 interaction is of high affinity.

### FGF-2 binding is confined to a small central region of the LTBP-2 molecule

**Fig 4:**
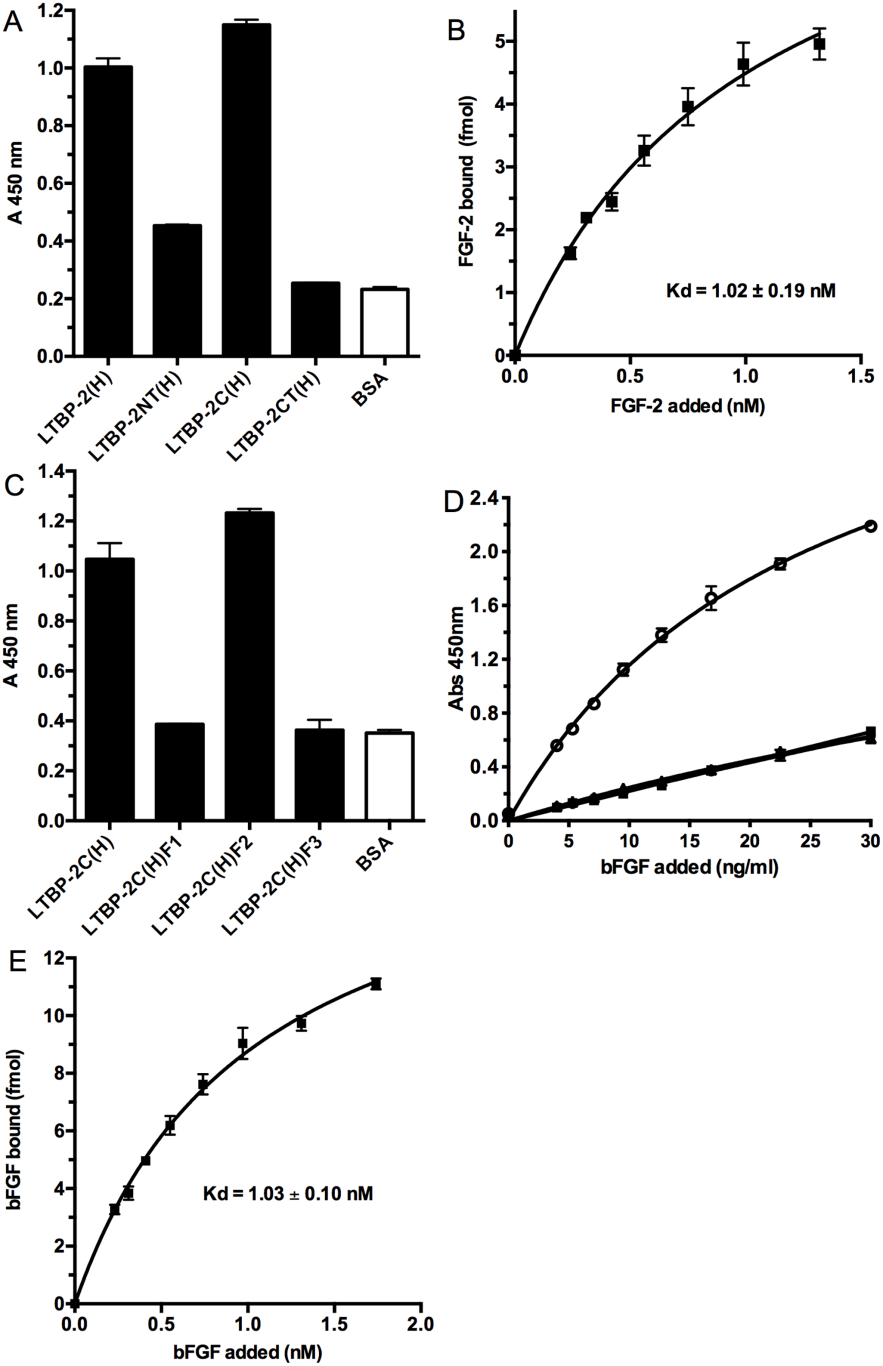
FGF-2 has a single binding domain in the central region of LTBP-2. **A**. Three recombinant fragments spanning the LTBP-2 molecule were tested for binding to FGF-2 in a solid phase assay. Full length LTBP-2(H), fragments LTBP-2 NT (H), LTBP-2C (H), LTBP-2 CT (H) or BSA control were coated onto wells at 100 ng/ml, followed by incubation with FGF-2 (100ng/ml) for 3h at 37°C. Strong specific binding to central fragment LTBP-2C(H) was detected as described in Fig 2A. Mean values ± S.D. from triplicate wells are shown. **B**. A binding curve was produced for the FGF-2 interaction with fragment LTBP-2C(H) following the protocol described under Fig 2, with 400 ng/well (4.8 pmol) of LTBP-2C (H) or BSA control coated on the wells incubated with increasing concentrations FGF-2 (0–1.5 nM). The Kd for binding of FGF-2 to fragment LTBP-2C (H) was calculated as 1.02 ± 0.19 nM. Mean values ± S.D. from triplicate determinations are shown. **C**. Three sub-fragments F1, F2 and F3 spanning fragment LTBP-2 C(H) were produced and tested for FGF-2 binding as described under Fig 2. LTBP-2C (H) (200 ng/well, 2.4 pmol) or sub-fragment (F1, F2 or F3) (66ng/well, 2.4 pmol) or BSA control was coated on the wells and incubated with FGF-2 (100 ng/ml). Strong specific binding of FGF-2 to sub-fragment LTBP-2C F2 was detected. Mean values ± S.D. from triplicate wells are shown. **D**. Subsequently binding curves were obtained for sub-fragments F1 (solid squares), F2 (open circles), F3 (solid circles) (35 ng/well, 1.2 pmol) coated on the wells and incubated with increasing concentrations of FGF-2 (0–30 ng / ml). Note specific FGF-2 binding to sub-fragment LTBP-2C F2 but no binding of fragments F1 and F3 above the BSA control (triangles). Mean values ± S.D. from triplicate determinations are shown. **E**. The Kd for the FGF-2 interaction with sub-fragment LTBP-2C F2 was calculated as 1.03 ± 0.10 nM which is similar to the Kds calculated for the interactions of FGF-2 with full-length LTBP-2 and fragment LTBP2C. Mean values ± S.D. from triplicate determinations are shown.

To identify the FGF-2 binding region(s) on LTBP-2, a range of recombinant LTBP-2 fragments were tested in the FGF-2 binding assay (Fig 4). Initially the three large fragments spanning the LTBP-2 molecule were tested with central fragment LTBP-2C(H) alone showing strong FGF-2 binding (Fig 4A). Subsequently three sub-fragments F1, F2, and F3 spanning LTBP-2C(H) were produced and tested with only the F2 showing strong FGF-2 binding (Fig 4C). This indicated that FGF-2 binding activity was confined to a small central region of LTBP-2 consisting of 6 calcium binding EGF-like repeat motifs (motifs 9–14) (see Fig 1A). Binding curves for fragment LTBP-2C(H) (not shown) and sub-fragment F2 (Fig 4D) were produced and the Kds for FGF-2 binding were calculated as previously for full length LTBP-2. The Kds for FGF-2 interaction with LTBP-2C(H) and F2 were calculated as 1.02 ± 0.19 nM (Fig 4B) and 1.03 ± 0.10 nM (Fig 4E) respectively indicating similar affinities for FGF-2 as the full-length LTBP-2 molecule. The binding affinity of LTBP-2 for FGF-2 was similar to that we reported for heparin [32] but was significantly higher than LTBP-2 interactions with fibrillin-1 (9 nM) and fibulin-5 (26 nM) using the same methodology [16, 17]. In an attempt to identify the precise binding site for FGF-2 on LTBP-2 we produced 6 peptides corresponding to each EGF-like motif of the FGF-2 binding region. However no direct FGF-2 binding or inhibition of the LTBP-2-FGF-2 interaction was identified for any of the peptides (data not shown). This indicates that the binding site may span two or more EGF-like repeats.

### The FGF-2 binding site is close to a heparin-binding region of LTBP-2

**Fig 5:**
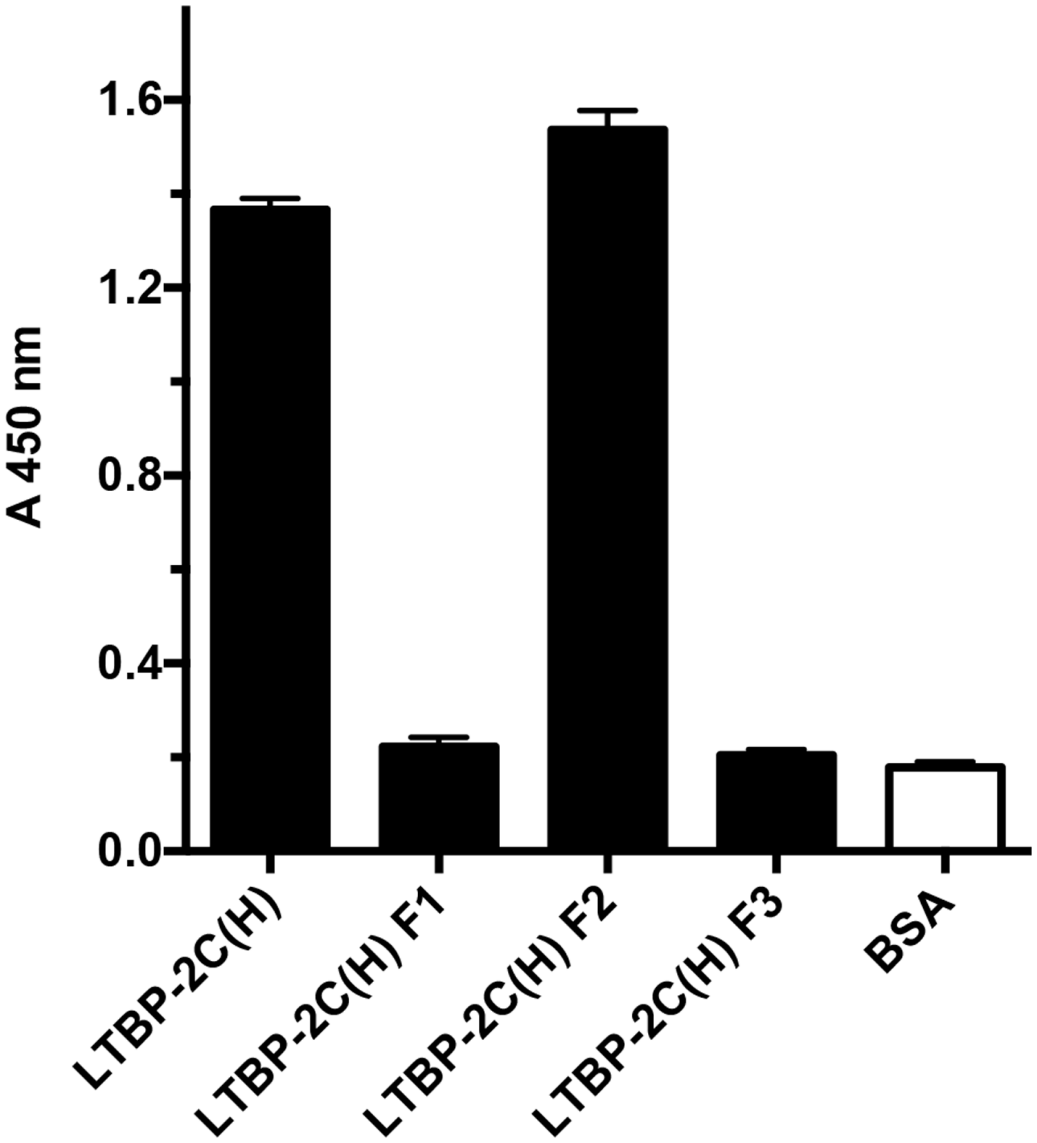
The FGF-2 binding site is close to the central heparin binding site on LTBP-2. In a previous study [32] we identified LTBP-2 C(H) as a heparin-binding fragment of LTBP-2. To further define the location of this heparin binding activity, the three sub-fragments F1, F2, F3 spanning LTBP-2 C(H), were assayed for heparin binding using a heparin-albumin conjugate (HAC). HAC or BSA control (400 ng) was coated on wells followed by incubation with equimolar concentrations (23.5 nM) of LTBP-2C(H) or sub-fragment F1, F2 or F3. Specific binding was detected using anti-His_4_ antibody targeting the poly-His tag on each recombinant fragment. Fragment F2 showed strong specific binding to the heparin conjugate in contrast to F1 and F3 which showed no binding above background. Mean values ± S.D. from triplicate wells are shown.

We have previously identified several heparin-binding regions on LTBP-2 including a central site of moderate affinity contained in fragment LTBP-2C(H) [32]. Since FGF-2 also has affinity for heparin/heparan sulphate we determined if the FGF-2 and heparin binding sites were contained in the same or distinct sub-fragments of LTBP-2C(H). Using the solid phase binding assay, fragments LTBP-2C(H) and sub-fragment F2 showed strong binding to heparin-albumin conjugate coated wells, whereas sub-fragments showed no binding above the control wells coated with BSA (Fig 5). Thus both the central heparin binding region and the FGF-2 binding site on LTBP-2 are present within six EGF-like repeats of each other. This site was reported to have moderate affinity for heparin with a Kd estimated at 80 nM compared to a cluster of higher affinity sites identified in the N-terminal region of LTBP-2 [32].

### LTBP-2 blocks FGF-2-induced cell proliferation

**Fig 6:**
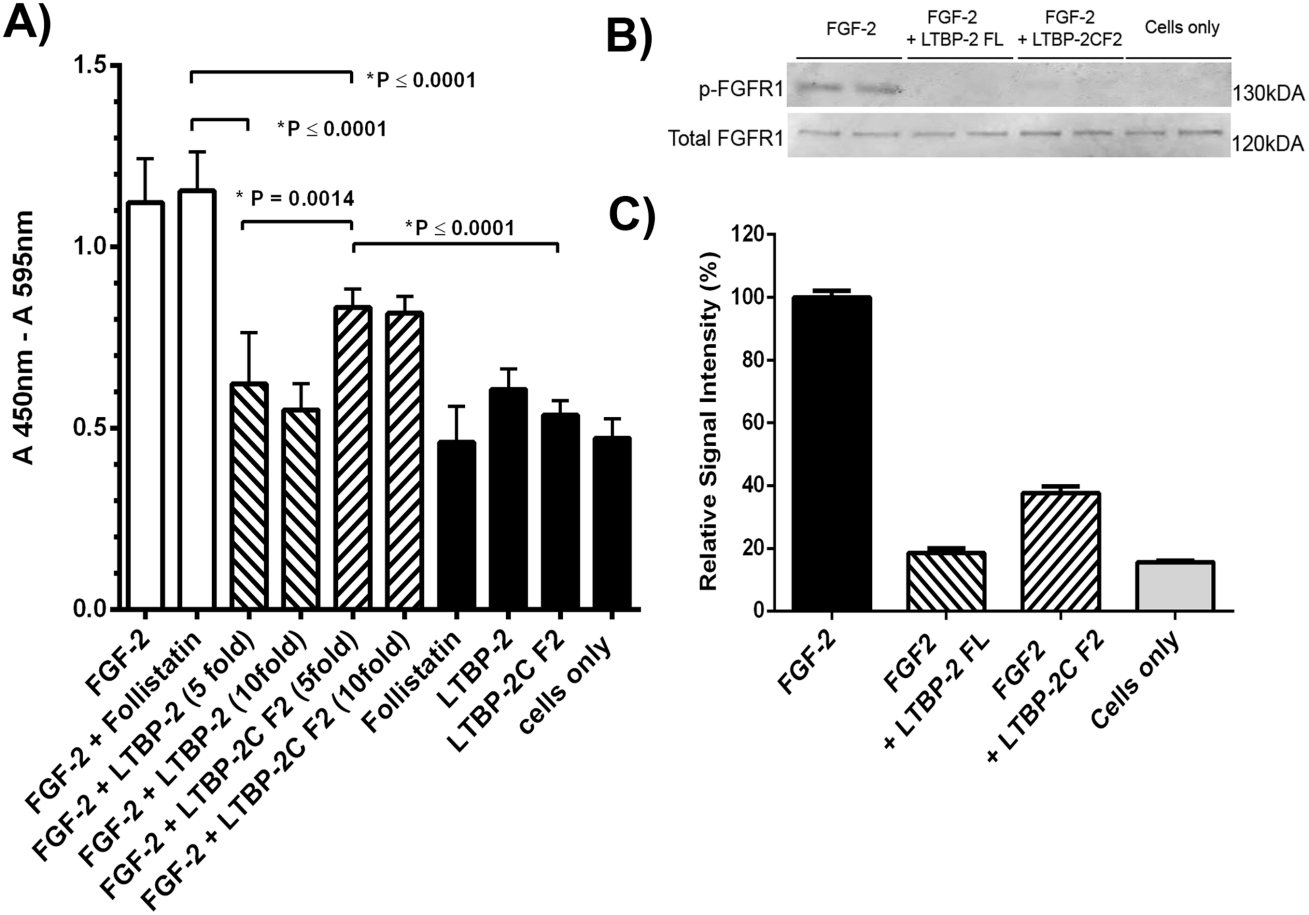
LTBP-2 blocks FGF-2-induced cell proliferation. **A**. The effect of LTBP-2 on the bio-activity of FGF-2 was tested in a cell proliferation assay (see experimental). Human foreskin fibroblasts were treated with FGF-2 with and without follistatin (white columns), or FGF-2 and follistatin pre-incubated with 5 or 10 fold molar excess of full length LTBP-2 or fragment LTBP-2C F2 (cross-hatched). Negative controls (black columns), included cells only and cells incubated with follistatin, LTBP-2 or fragment LTBP-2C F2. Mean values ± S.D. from triplicate determinations. Note 5 fold molar excess of full-length LTBP-2 completely blocked FGF-2 induced cell proliferation (p = 0.0001) and 5-fold molar excess of fragment LTBP-2C F2 partially blocked the activity (p = 0.0001). **B**. Immunoblot analysis FGF receptor (FGFR1) phosphorylation. Human foreskin fibroblasts were treated for 2 hours with FGF-2 (10 ng / ml) only or with FGF-2 plus 10-fold molar excess of full length LTBP-2 (LTBP-2 FL) or fragment F2 (LTBP-2C F2). Control cells had no FGF-2 or LTBP-2 added. Cellular proteins were extracted and duplicate samples were analysed by SDS-PAGE and immunoblotting with anti-phospho-FGFR1 antibody, and anti-total FGFR1 antibody. Bands were visualised using the LI-COR Odyssey Infrared Imaging System. **C**. The band intensity was measured using ImageJ 1.48 software [National Institutes of Health (NIH), Bethesda, MD] and normalised to the internal β actin signal. The ratio of the phospho-FGFR1 to total FGFR1 value for each sample is expressed relative to the average FGF-2 only control value (= 100%). Note the strong FGFR1 activation by FGF-2 was substantially blocked by both LTBP-2 C and LTBP-2C F2 fragments. Mean values ± S.D. of duplicate lanes.

To determine if LTBP-2 enhanced or inhibited FGF-2 bioactivity a cell proliferation assay was conducted (Fig 6A). Addition of exogenous FGF-2 was found to significantly increase the rate of proliferation of fibroblasts in serum-free culture over 48 h, to a similar extent in the presence or absence of activin/TGF-β inhibitor follistatin. However pre-incubation of the FGF-2 with full-length LTBP-2 in 5-fold or 10-fold molar excess prevented any FGF-2-induced cell proliferation. Pre-incubation with fragment LTBP-2C-F2, which contains the FGF-2 binding site, also significantly inhibited, but did not completely block, FGF-2 induced cell proliferation. Controls conducted in the absence of FGF-2 showed that follistatin, LTBP-2 or fragment LTBP-2C F2 had no significant effect on cell proliferation. To determine if LTBP-2 blocked the activation of the FGF receptor, the experiment was repeated and cellular proteins were extracted after 2 hours and analysed by SDS-PAGE and immunoblotting (Fig 6B and 6C). The results clearly showed that the control cells had no detectable activated FGFR1 but the addition of FGF-2 resulted in a strong FGFR1 signal. Additional of excess full length LTBP-2 completely blocked the activation of the receptor but the same molarity of fragment LTBP-2CF2 greatly reduced but did not completely prevent FGFR1 activation. Overall the experiment indicated that LTBP-2 inhibits rather than enhances FGF-2 activity. It is noteworthy that the 6-EGF-like repeat fragment containing the FGF-2 binding sequence (LTBP-2C F2) only partially inhibited the mitogenic effect of FGF-2. Thus additional sequences adjacent to fragment F2 may be important for the full influence of LTBP-2 on FGF-2 bioactivity.

### LTBP-2 and FGF-2 show similar distributions in fibrotic skin

**Fig 7:**
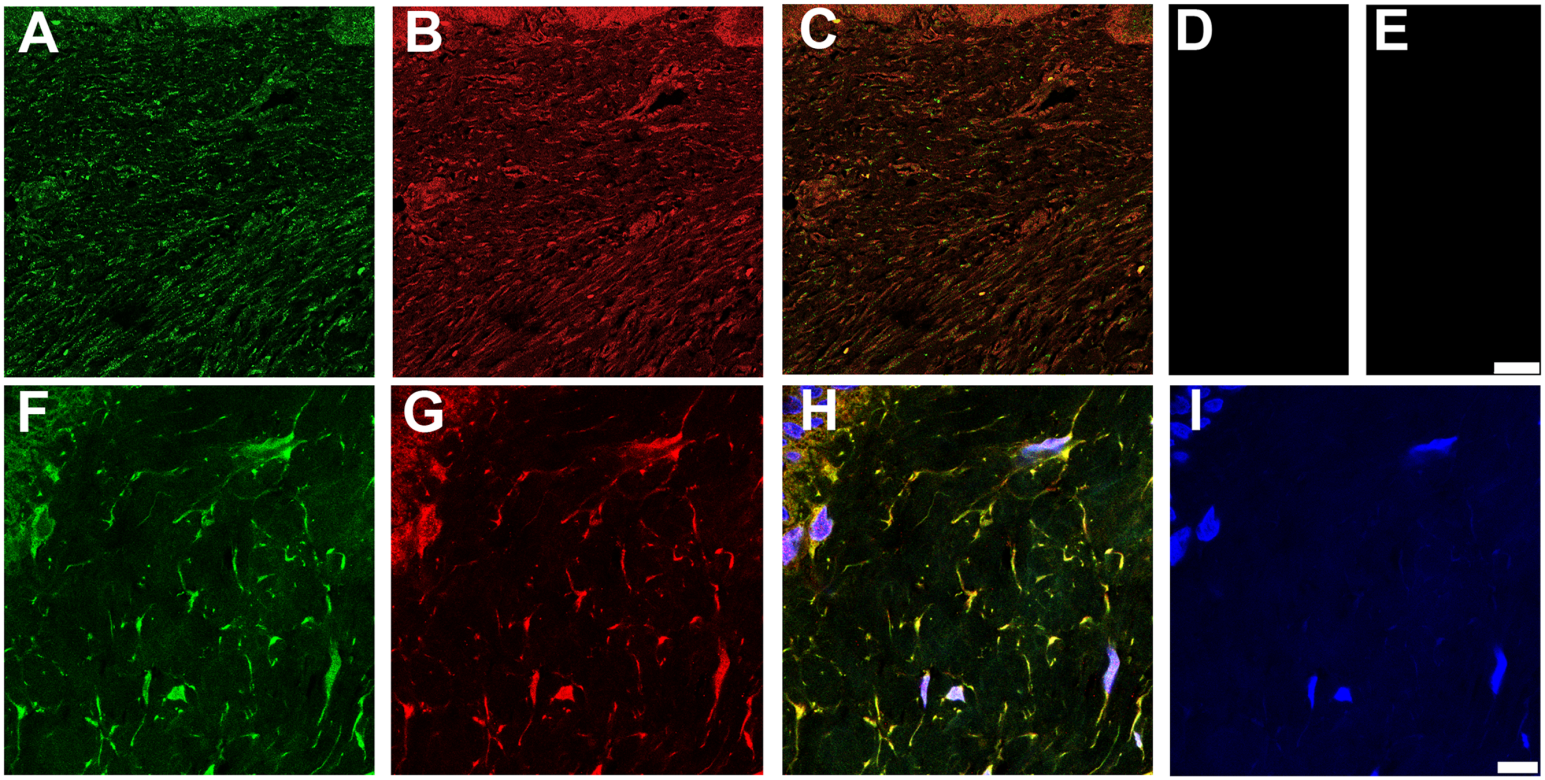
LTBP-2 and fibrillin-1 colocalize in fibrotic skin. Keloid tissue was prepared and analyzed by confocal microscopy as described in the methods section. **A and F**, polyclonal anti-[human LTBP-2 peptide] antibody 3504 (2 μg/ ml) detected with anti-rabbit IgG antibody conjugated to fluor Alexa 488; **B and G**, monoclonal anti-[fibrillin-1] antibody MAB1919 (Merck millipore) (2.5 μg/ml) detected with anti-mouse IgG antibody conjugated to Alexa 594; **C**, A and B merged; **D**, rabbit IgG control (2 μg/ ml); **E**, mouse IgG control (2.5 μg / ml); **H**, F, G and I merged; **I**, DAPI nuclear stain. Magnification: top row, Bar = 100 μm; bottom row, Bar = 10 μm.

**Fig 8:**
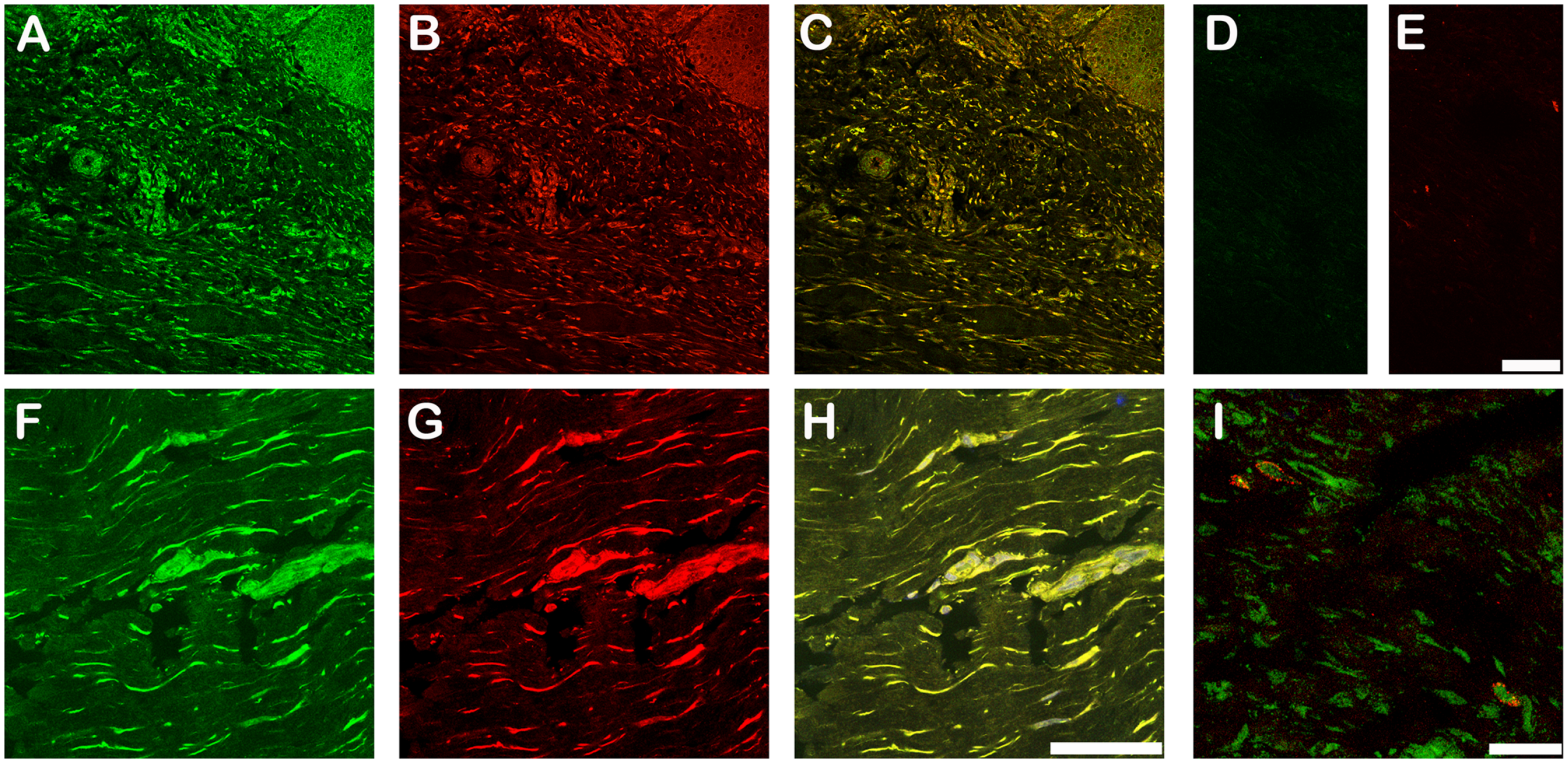
LTBP-2 and FGF-2 co-localize in keloid tissue. Keloid tissue was also analyzed for LTBP-2 and FGF-2 by confocal microscopy. **A and F**, polyclonal anti-[human LTBP-2 peptide] antibody 3504 (2 μg/ ml) detected with anti-rabbit IgG antibody conjugated to fluor Alexa 488; **B and G**, monoclonal anti-[human FGF-2] antibody #61087 (BD Biosciences) (2.5 μg/ml) detected with anti-mouse IgG antibody conjugated to Alexa 594; **C**, A and B merged; **D**, rabbit IgG control (2 μg/ ml); **E**, mouse IgG control (2.5 μg / ml); **H**, F, and G merged; **I**, Control confocal image showing distinct immunostaining patterns for VEGF (red) and LTBP-2 (green). Magnification: top row, Bar = 100 μm; bottom row, Bar = 50 μm.

**Fig 9:**
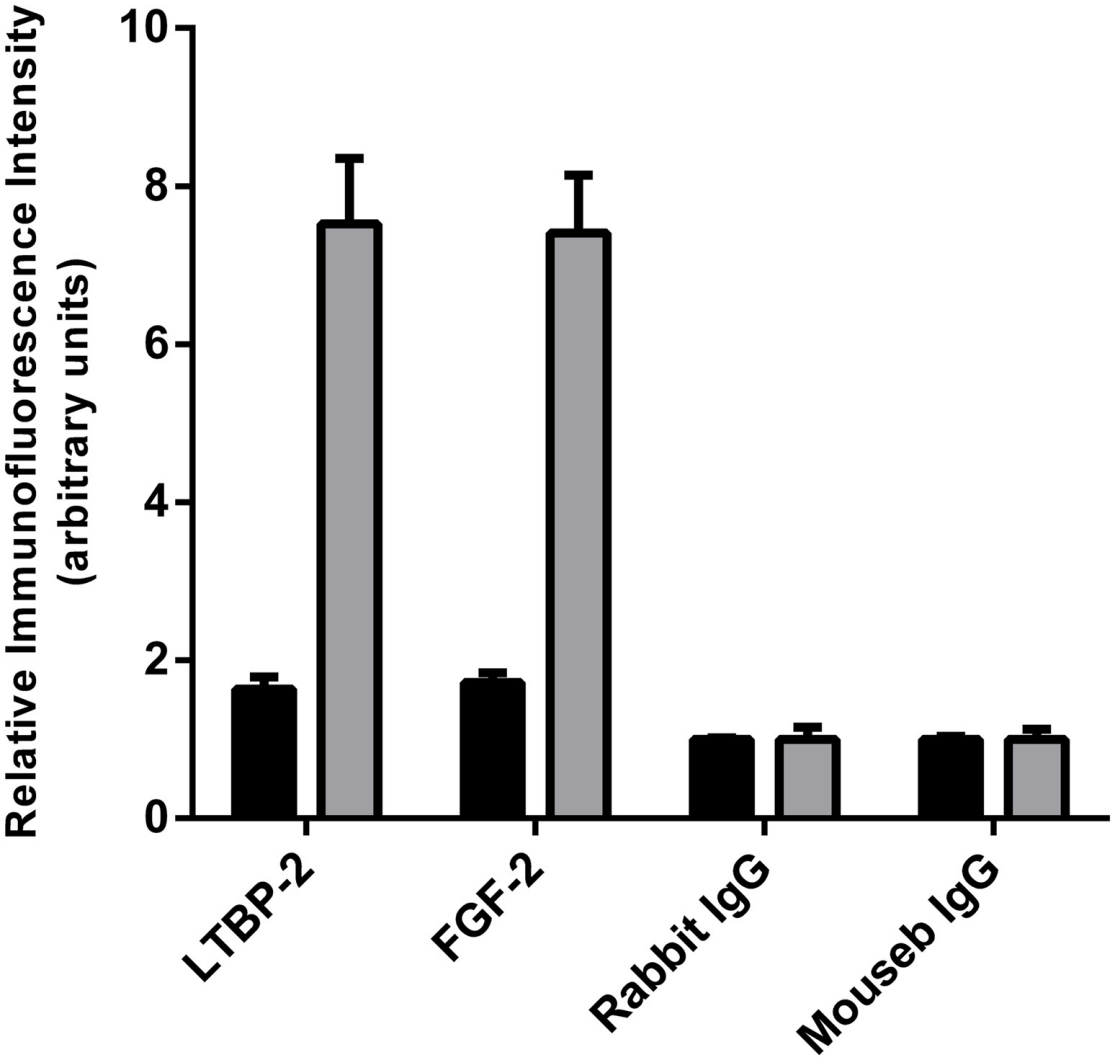
Quantitation of LTBP-2 and FGF-2 in normal skin and keloid. The relative fluorescence intensities of LTBP-2 and FGF-2 staining (and appropriate IgG controls) in sections of normal human skin (black columns) and keloid (shaded columns) was quantitated from 3 random areas (each 0.038 mm^2^) per section using the AnalySIS software package (Soft-Imaging System, Munster, Germany). Values expressed relative to the background control signal (= 1 unit). Mean values ± S.D. of triplicate determination are shown.

To determine if the interaction of LTBP-2 and FGF-2 could have biological relevance we searched for overlapping of immunofluorescence localization patterns in normal and fibrotic skin. Neither protein showed discernible localization within the extracellular matrix of normal adult skin (data not shown). LTBP-2 gene mutations have been linked to WMS which demonstrates thickened fibrotic skin suggesting a connection between LTBP-2 and fibrosis [18]. We therefore examined LTBP-2 expression in fibrotic keloid tissue that has elevated production of new elastic fibres [37, 38]. Keloids are fibrotic scars that are raised above skin level and project beyond the original wound margins [39]. The keloid tissue stained very strongly for LTBP-2 with a widespread, fibrous distribution (Fig 7A) which closely matched the distribution of fibrillin-1 (Fig 7B) as confirmed by the merged images (Fig 7C). Control sections incubated with rabbit or mouse IgG in place of antibody showed no discernible staining (Fig 7D and 7E). At high power, fine irregular fibres staining for both LTBP-2 (Fig 7F) and fibrillin-1 (Fig 7G) were evident in the intercellular matrix, visualized as yellow staining in the merged image (Fig 7H). The results indicate that LTBP-2 is predominantly associated with fibrillin-containing microfibrils, which are components of elastic fibres. These findings are consistent with previous studies showing strong co-localization of LTBP-2 and developing elastin fibres in fetal tissues and in tissue remodelling [8, 10, 40]. The elastic fibres generally ran parallel to the epithelium although some areas showed a more random distribution consistent with previous reports [37, 38]. Interestingly a similar intense immuno-staining pattern was found for FGF-2 in sections of fibrotic keloid skin from several patients. An example from one patient is shown in Fig 7. Low power images show intense discrete staining for LTBP-2 (Fig 8A-green) and FGF-2 (Fig 8B-red) to the same structures throughout the keloid as confirmed from the merged image (Fig 8C) where co-localization is visualized as yellow-orange. At higher power, LTBP-2 (Fig 8F-green) and FGF-2 (Fig 8G-red) antibodies stained the same fibres within the extracellular matrix as well as cellular elements (identified using the blue nuclear DAPI stain). The extensive overlap of staining for the two proteins is confirmed by the merged image (Fig 8H) where the co-localization is visualized as yellow staining. The appropriate immunoglobulin controls showed little background staining (Fig 8D and 8E). As an additional control a section was stained for LTBP-2 and VEGF which has no known affinity for fibrillin microfibrils (Fig 8I). No overlap in the distributions were observed, with VEGF detected only in association with some but not all of the stromal cells and showing no localization within the extracellular matrix. The close proximity of FGF-2 to LTBP-2 within the keloid indicates that the two proteins may directly interact in the matrix of fibrotic skin on the surface of newly generated elastic fibres where they may influence, in vivo, the biological activity of each other. The significance of the strong intracellular staining for both proteins is less clear. It seems likely that this simply reflects high synthesis rates for both proteins in fibrotic tissues although a direct intracellular interaction cannot be ruled out. Quantitation of the relative immunofluorescence signals between normal skin and keloid showed around 9-fold increases in signals for both LTBP-2 and FGF-2 in the keloid tissue suggesting that production of both proteins was greatly increased in the fibrotic condition (Fig 9).

Our results have shown that LTBP-2 strongly binds and inactivates FGF-2 in vitro and that both proteins appear to co-localize with fibrillin-microfibrils in fibrotic tissues. However the importance of these observations in microfibril and elastic fibre biology, and pathophysiology of relevant congenital and fibrotic diseases, remains to be established. The paradigm of the congenital disease MFS and related disorders has demonstrated that fibrillin microfibrils are important for growth factor regulation. Mutations in fibrillin genes cause a reduction in the number of normal microfibrils in tissues, resulting in inappropriate or excessive activation of latent TGF-β during tissue development and growth [7, 20]. This aberrant TGF-β signaling is considered to be a major contributor to the malformation and dysfunction of the cardiovascular, skeletal, pulmonary and ocular systems characteristic of MFS. The mechanism of this TGF-β activation appears to be more complex than originally envisaged. Isogai et al showed that LTBP-1, 3 and 4 share a single binding site on fibrillin-1 and suggested that disruption of this binding activity would reduce matrix storage of the LTBP-TGF-β latent complexes resulting in excessive growth factor activation [41]. However subsequent research with mutant mice showed that total deletion of this binding site on fibrillin-1 caused no obvious disease phenotype [42]. More recently Zilberberg et al demonstrated that LTBP-1, the major contributor to latent TGF-β sequestration, required only fibronectin and not fibrillin 1 or 2 for matrix attachment [43]. The findings suggest that other mechanisms in addition to direct liberation of latent TGF-β from the fibrillin microfibrils may contribute to elevation of the TGF-β signalling. Since fibrillin and associated proteins also bind a range of other potent cytokines, it seems likely that disruption of normal microfibrils will activate other signalling pathways perhaps leading to indirect TGF-β elevation. It appears that LTBP-2 requires fibrillin-1 microfibrils for incorporation into the extracellular matrix [44] and thus loss of these structures is likely to disrupt matrix sequestration of LTBP-2 and any attached proteins such as FGF-2. Depending on context FGF-2 can stimulate TGF-ß gene expression [45] and secretion [46] or can inhibit TGF-β induced fibrosis [31]. Thus it is difficult to predict possible effects of disrupting LTBP-2/FGF-2 interactions in WMS and other relevant diseases. The LTBP-2 gene has also been linked to tumor suppression in squamous cell carcinoma and meningioma, [47, 48] and as a marker for pulmonary deaths following acute dyspnea [49]

It also remains to be established how LTBP-2 relates to FGF-2 functional biology. FGF-2 lacks a secretion signal [50] and is secreted from cells by an unknown mechanism and becomes strongly bound to the GAG side-chains of HSPGs in the matrix and basement membranes [51, 52] Following tissue injury, the FGF- 2 molecules are released by protease and heparinase activity. Multiple FGF-2 molecules remain attached to released HS chains and subsequent interaction with cell surface FGF-receptors causes clustering of the FGFR molecules necessary to activate intracellular signaling pathways [51, 52]. FGF-2 achieves its diverse effects by stimulating several major cell signaling pathways including RAS/MAPK, PI3K/AKT and PLC-ϒ [53] and in complex with cell surface heparan sulphate proteoglycans, the ERK1/2 pathway [54].

We have shown here that the FGF-2 binding site of LTBP-2 is adjacent to a heparin binding site of moderate affinity. LTBP-2 also has multiple high affinity binding sites for heparin/heparan sulfate in its N-terminal region, binds HSPGs perlecan in vitro [32] and partially co-localizes with the proteoglycan in some tissues [55, 56]. The findings suggest that LTBP-2, in addition to free FGF-2, may also target and inhibit heparan sulphate-bound growth factor. Interestingly, Cain et al. have recently shown that fibrillin-1 interactions with heparan sulfate may be disrupted in WMS [57] and it is possible that LTBP-2 interactions with FGF-2 and heparan sulfate are affected in WMS cases linked to LTBP-2 gene mutations.

The association of LTBP-2 with elastic fibres is well documented during periods of active elastinogenesis [8, 40] but the protein is not ubiquitously associated with all elastic fibres [17]. This restriction may explain why FGF-2 localization to elastic fibres has not previously been reported since its association may be dependent on the presence of LTBP-2. The high levels of LTBP-2 in keloid tissue suggests a potential role for the protein in fibrosis. FGF-2 has an anti-fibrotic role in the later stages of wound healing and exogenous FGF-2 has been used to effect in treatment of hypertrophic scar and keloid tissues [31, 58]. An intriguing possibility is that in keloid and perhaps other fibrotic disorders elevated LTBP-2 may bind and inactivate FGF-2, inhibiting its contribution to resolution and healing of the condition and perpetuating the fibrotic process. This suggestion warrants further investigation.

## Supporting Information

S1 Raw Data (ZIP)
