# Supplementary material for: LTBP-2 Has a Single High-Affinity Binding Site for FGF-2 and Blocks FGF-2-Induced Cell Proliferation
Source: PLoS One. 2015 Aug 11;10(8):e0135577. doi: 10.1371/journal.pone.0135577 (PMC4532469; doi:10.1371/journal.pone.0135577)
Supplement: S1 Raw Data — (ZIP) [file pone.0135577.s001.zip › supporting information resubmission 2/Fig 2/Fig 2A Raw Data.pdf]

	LTBP-2			BSA		
TGF-beta	0.254	0.259	0.265	0.223	0.239	0.253
VEFG	0.208	0.198	0.201	0.164	0.184	0.154
BMP-7	0.195	0.191	0.186	0.181	0.174	0.175
BMP-4	0.397	0.390	0.381	0.187	0.175	0.168
FGF-2	0.490	0.493	0.495	0.180	0.173	0.167

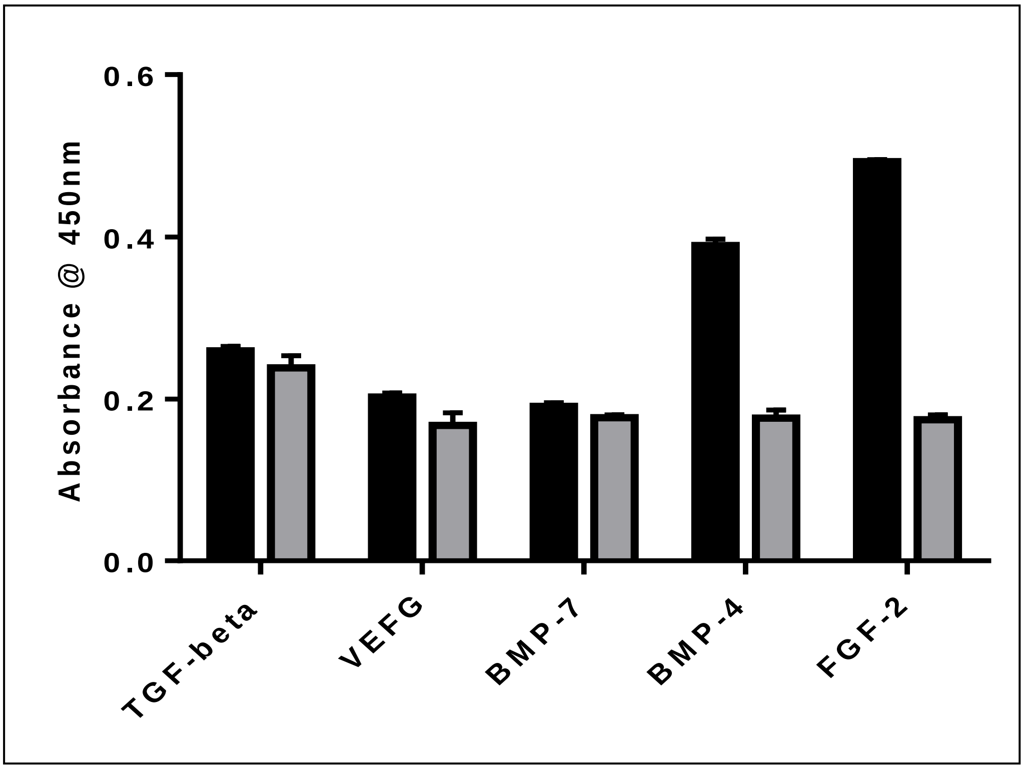

Figure 2. LTBP-2 specifically binds FGF-2 but not VEGF, BMP-4, BMP-7 or TGF-beta
A. Microtitre wells were coated with rLTBP-2 (black columns) or BSA(shaded columns) (100 ng/ well). After blocking, triplicate wells were incubated at 37°C for 2h with TGF-beta (13 ng / well), VEGF (21 ng / well), BMP-7 (4 ng/well), BMP-4 (4 ng / well) or FGF-2 (10 ng / well). Growth factor binding was detected using specific biotinylated antibodies from Duoset kits as described in material and methods. Mean values ± S.D. from triplicate wells are shown.
