# Supplementary material for: LTBP-2 Has a Single High-Affinity Binding Site for FGF-2 and Blocks FGF-2-Induced Cell Proliferation
Source: PLoS One. 2015 Aug 11;10(8):e0135577. doi: 10.1371/journal.pone.0135577 (PMC4532469; doi:10.1371/journal.pone.0135577)
Supplement: S1 Raw Data — (ZIP) [file pone.0135577.s001.zip › supporting information resubmission 2/Fig 2/Fig 2B Raw Data.pdf]

| | | | | | | |
|---|---|---|---|---|---|---|
| LTBP-2 | BMP-4 + | | | BMP-4 - | | |
| | 0.384 | 0.402 | 0.394 | 0.400 | 0.382 | 0.349 |
| LTBP-2 | FGF-2 + | | | FGF-2 - | | |
| | 0.490 | 0.493 | 0.495 | 0.180 | 0.174 | 0.168 |

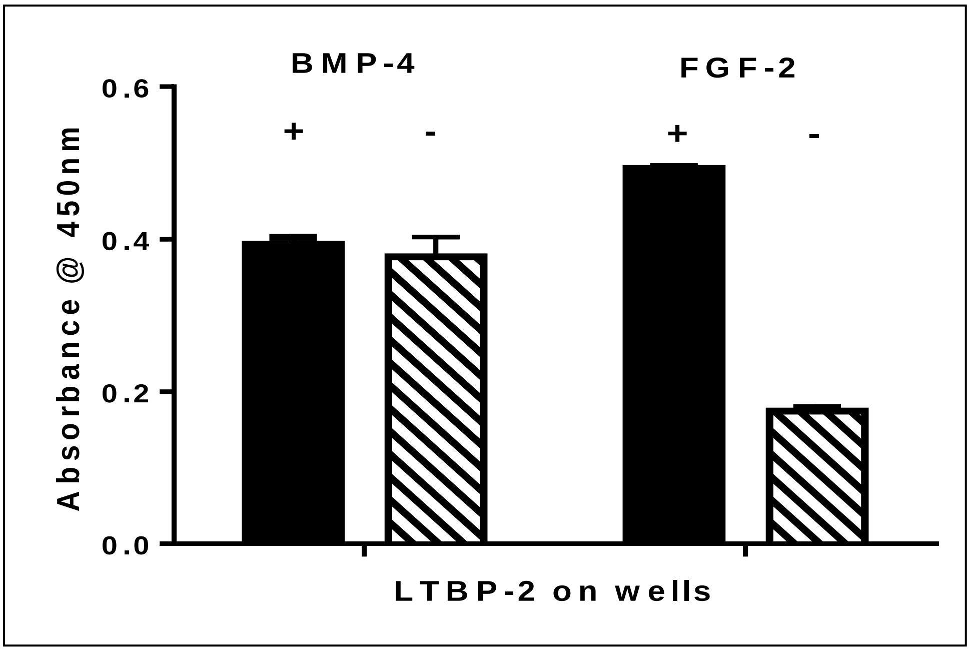


B. Microtitre wells were coated with rLTBP-2 (100ng/well) was coated onto microtitre plates. After blocking, triplicate wells were incubated at 37$^{o}$C for 2h with (black columns) or without (cross-hatched) growth factor, (BMP-4 (4ng/well) or FGF-2 (10ng/well). Binding of growth factor to LTBP-2 was detected using biotinylated anti-BMP-4 detection antibody (0.5ug/ml) or anti-FGF-2 detection antibody (0.25ug/ml), followed by a peroxidase detection method (see material and methods). Mean values ± S.D. from triplicate wells are shown. Note the anti-BMP-4 antibody bound to the wells equally strongly in the presence or absence of added BMP-4, indicating the interaction was non-specific.
