# Supplementary material for: LTBP-2 Has a Single High-Affinity Binding Site for FGF-2 and Blocks FGF-2-Induced Cell Proliferation
Source: PLoS One. 2015 Aug 11;10(8):e0135577. doi: 10.1371/journal.pone.0135577 (PMC4532469; doi:10.1371/journal.pone.0135577)
Supplement: S1 Raw Data — (ZIP) [file pone.0135577.s001.zip › supporting information resubmission 2/Fig 3/Fig 3A Raw Data.pdf]

bFGF added ng/ml	LTBP-2FL			BSA		
0.0	0.000	0.000	0.000	0.000	0.000	0.000
4.0	0.277	0.134	0.164	0.021	0.022	0.014
5.3	0.201	0.226	0.227	0.051	0.017	0.058
7.1	0.282	0.276	0.285	0.048	0.038	0.037
9.5	0.360	0.285	0.414	0.098	0.066	0.091
12.7	0.443	0.381	0.433	0.093	0.088	0.072
16.8	0.417	0.516	0.521	0.166	0.122	0.129
22.5	0.700	0.698	0.652	0.209	0.206	0.172
30.0	0.714	0.779	0.772	0.306	0.243	0.241

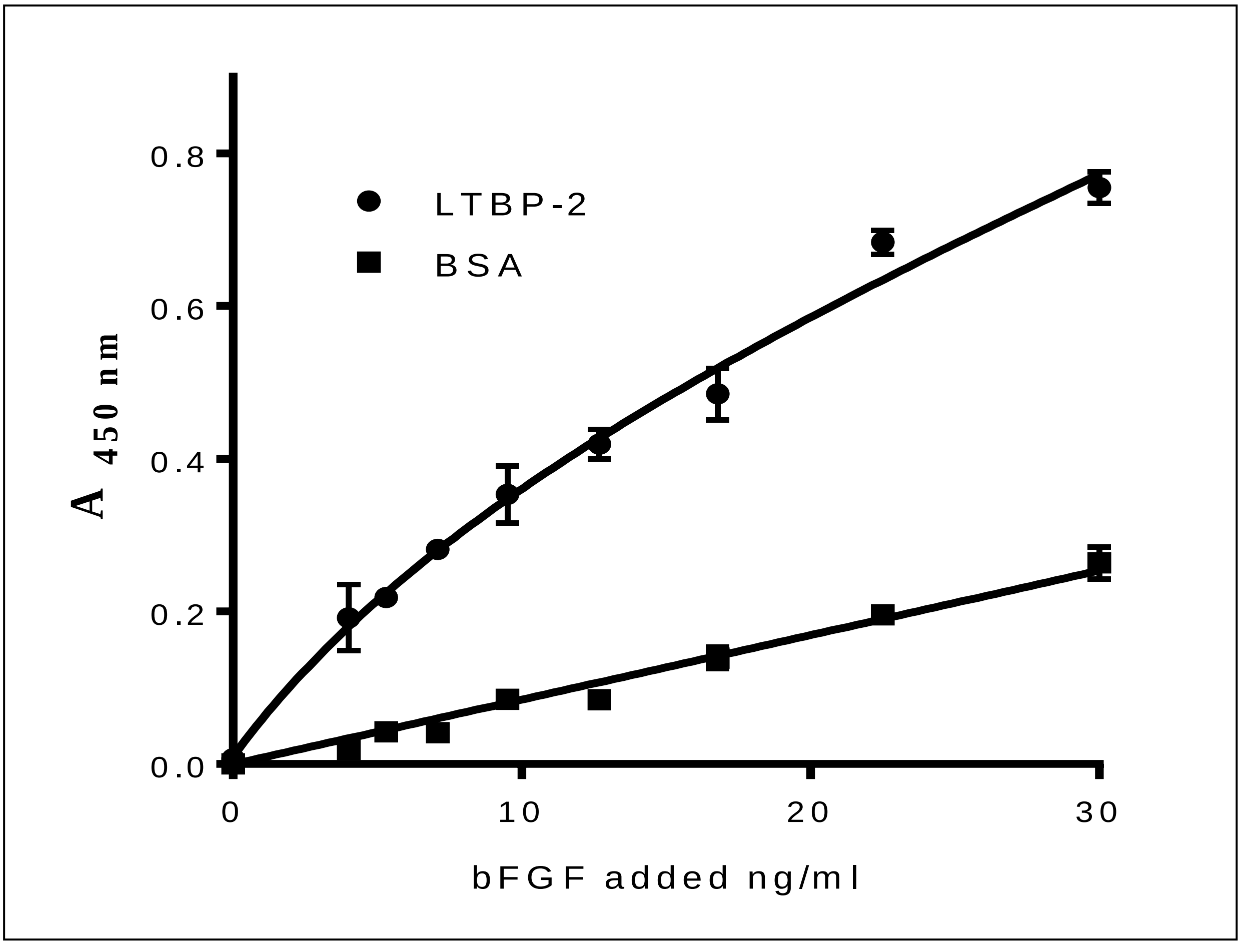

Figure 3. LTBP-2 interacts strongly with FGF-2. **A).** Microtitre wells were coated with 200 ng rLTBP-2 or BSA control. After blocking, triplicate wells were incubated with 0- 1.8 nM concentrations of FGF-2 (0- 30 ng/ml) for 3 h at 37°C. FGF-2 binding was detected following sequential incubation of the wells with biotinylated mouse anti-[human FGF-2] antibody and streptavidin-HRP conjugate following the duoset protocol. Circles, LTBP-2; squares, BSA
