# Supplementary material for: LTBP-2 Has a Single High-Affinity Binding Site for FGF-2 and Blocks FGF-2-Induced Cell Proliferation
Source: PLoS One. 2015 Aug 11;10(8):e0135577. doi: 10.1371/journal.pone.0135577 (PMC4532469; doi:10.1371/journal.pone.0135577)
Supplement: S1 Raw Data — (ZIP) [file pone.0135577.s001.zip › supporting information resubmission 2/Fig 3/Fig 3B Raw Data.pdf]

bFGF added nM	bFGF bound (fmol)		
0.00	0.00	0.00	0.00
0.24	0.27	0.28	0.34
0.31	0.36	0.40	0.44
0.42	0.51	0.54	0.52
0.56	0.63	0.44	0.72
0.75	0.81	0.66	0.77
0.99	0.84	0.83	0.86
1.32	1.09	1.11	1.02
1.76	0.99	1.15	1.11

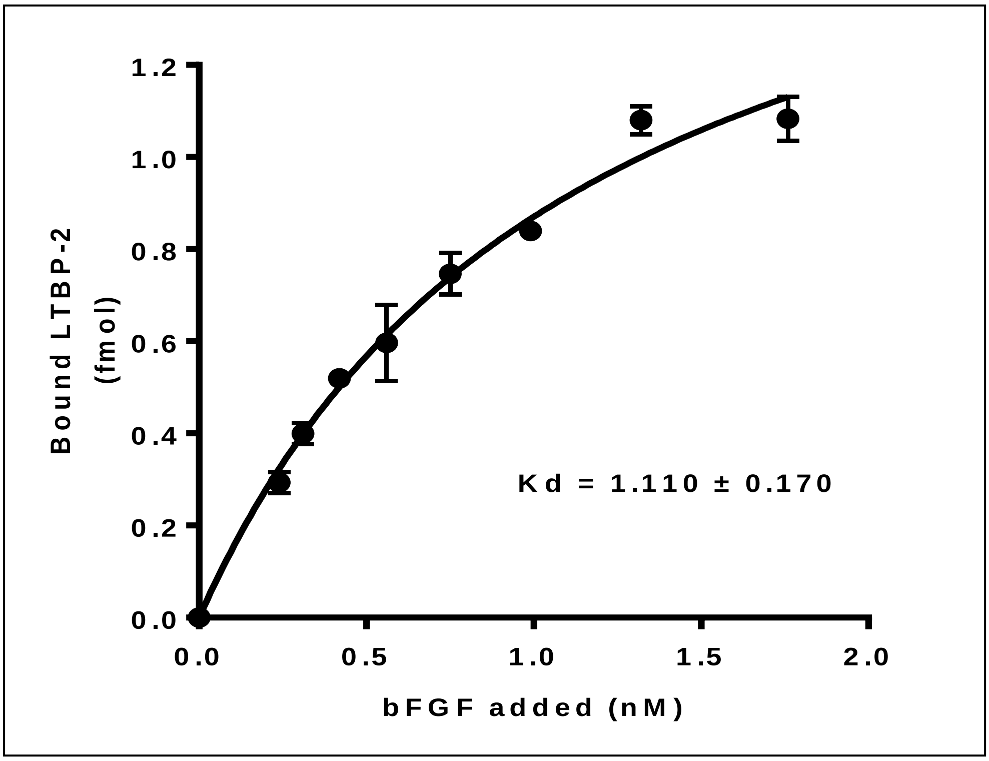

B). Kd calculation. Following subtraction of the average BSA signal, the A450nm values were converted to fmol of FGF-2 using a standard ELISA curve (not shown). An additional graph was plotted of bound versus added FGF-2 and the Kd for interaction with LTBP-2 was calculated by non-linear regression analysis of the curve using the prism 4.0 program.
