# Supplementary material for: LTBP-2 Has a Single High-Affinity Binding Site for FGF-2 and Blocks FGF-2-Induced Cell Proliferation
Source: PLoS One. 2015 Aug 11;10(8):e0135577. doi: 10.1371/journal.pone.0135577 (PMC4532469; doi:10.1371/journal.pone.0135577)
Supplement: S1 Raw Data — (ZIP) [file pone.0135577.s001.zip › supporting information resubmission 2/Fig 4/Fig 4A.pdf]

| LTBP-2(H) | LTBP-2NT(H) | LTBP-2C(H) | LTBP-2CT(H) | BSA |
|---|---|---|---|---|
| 1.051 | 0.453 | 1.174 | 0.254 | 0.237 |
| 1.011 | 0.447 | 1.114 | 0.256 | 0.216 |
| 0.949 | 0.460 | 1.161 | 0.251 | 0.244 |

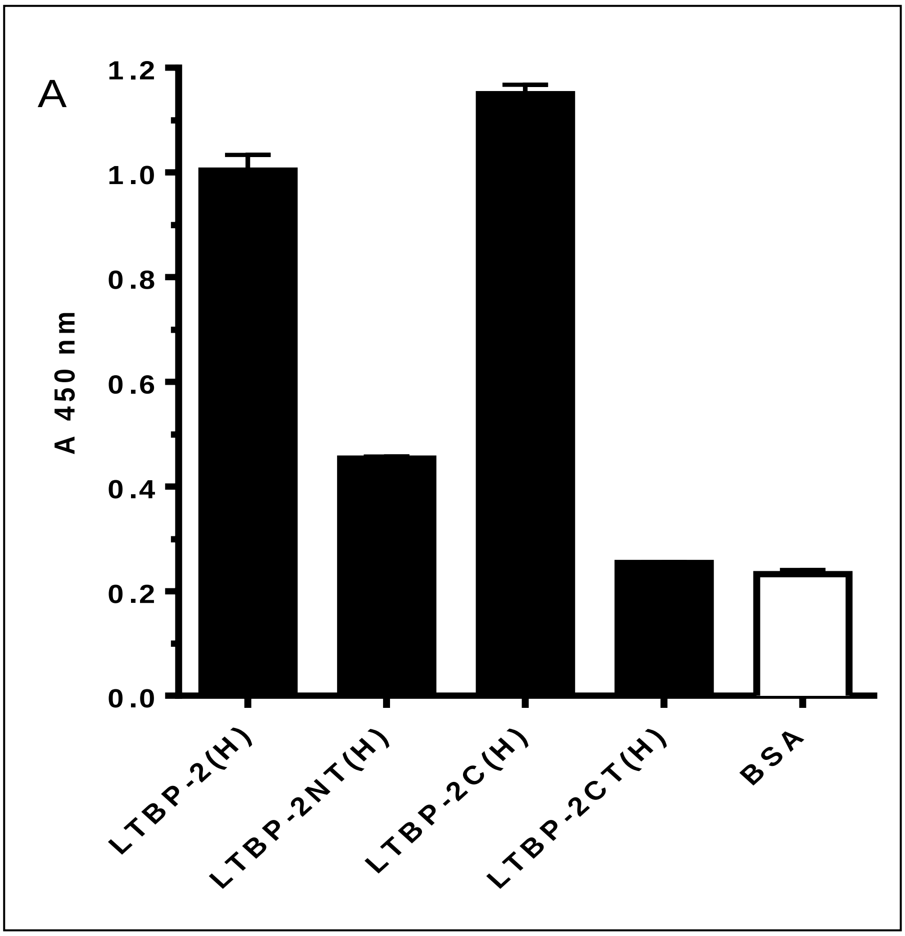


**Figure 4. FGF-2 has a single binding domain in the central region of LTBP-2.**
**A).** Three recombinant fragments spanning the LTBP-2 molecule were tested for binding to FGF-2 in a solid phase assay. Full length LTBP-2(H), fragments LTBP-2 NT (H), LTBP-2C (H), LTBP-2 CT (H) or BSA control were coated onto wells at 100 ng/ml, followed by incubation with FGF-2 (100ng/ml) for 3h at 37 °C. Strong specific binding to central fragment LTBP-2C(H) was detected as described in Fig 2A.
