# Supplementary material for: LTBP-2 Has a Single High-Affinity Binding Site for FGF-2 and Blocks FGF-2-Induced Cell Proliferation
Source: PLoS One. 2015 Aug 11;10(8):e0135577. doi: 10.1371/journal.pone.0135577 (PMC4532469; doi:10.1371/journal.pone.0135577)
Supplement: S1 Raw Data — (ZIP) [file pone.0135577.s001.zip › supporting information resubmission 2/Fig 4/Fig 4B.pdf]

bFGF nM added	bFGF fmol per nM Bound		
0.00	0.00	0.00	0.00
0.24	1.71	1.72	1.44
0.31	2.20	2.11	2.27
0.42	2.27	2.35	2.72
0.56	3.09	2.96	3.73
0.75	3.44	3.98	4.46
0.99	5.29	4.14	4.48
1.32	4.88	4.57	5.42

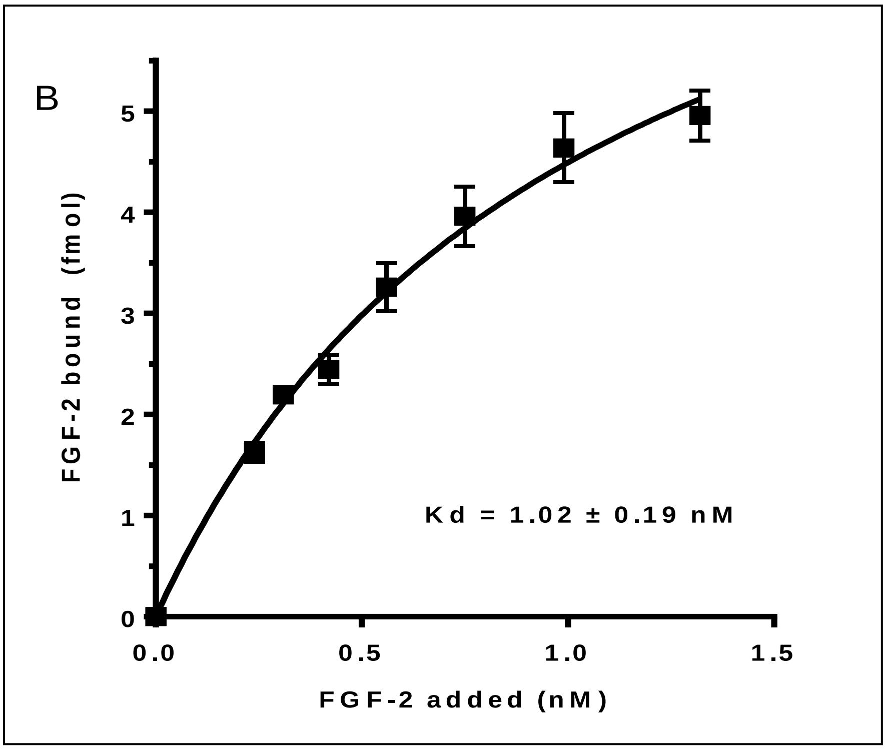

Figure 4. FGF-2 has a single binding domain in the central region of LTBP-2.

B). A binding curve was produced for the FGF-2 interaction with fragment LTBP-2C(H) following the protocol described under figure 2, with 400 ng/well (4.8 pmol) of LTBP-2C (H) or BSA control coated on the wells incubated with increasing concentrations FGF-2 (0- 1.5 nM). The Kd for binding of FGF-2 to fragment LTBP-2C (H) was calculated as 1.02 $\pm$ 0.19 nM.
