# Supplementary material for: LTBP-2 Has a Single High-Affinity Binding Site for FGF-2 and Blocks FGF-2-Induced Cell Proliferation
Source: PLoS One. 2015 Aug 11;10(8):e0135577. doi: 10.1371/journal.pone.0135577 (PMC4532469; doi:10.1371/journal.pone.0135577)
Supplement: S1 Raw Data — (ZIP) [file pone.0135577.s001.zip › supporting information resubmission 2/Fig 4/Fig 4C.pdf]

LTBP-2C(H)	LTBP-2C(H)F1	LTBP-2C(H)F2	LTBP-2C(H)F3	BSA
1.076	0.386	1.206	0.365	0.355
1.141	0.385	1.262	0.291	0.328
0.925	0.388	1.229	0.434	0.371

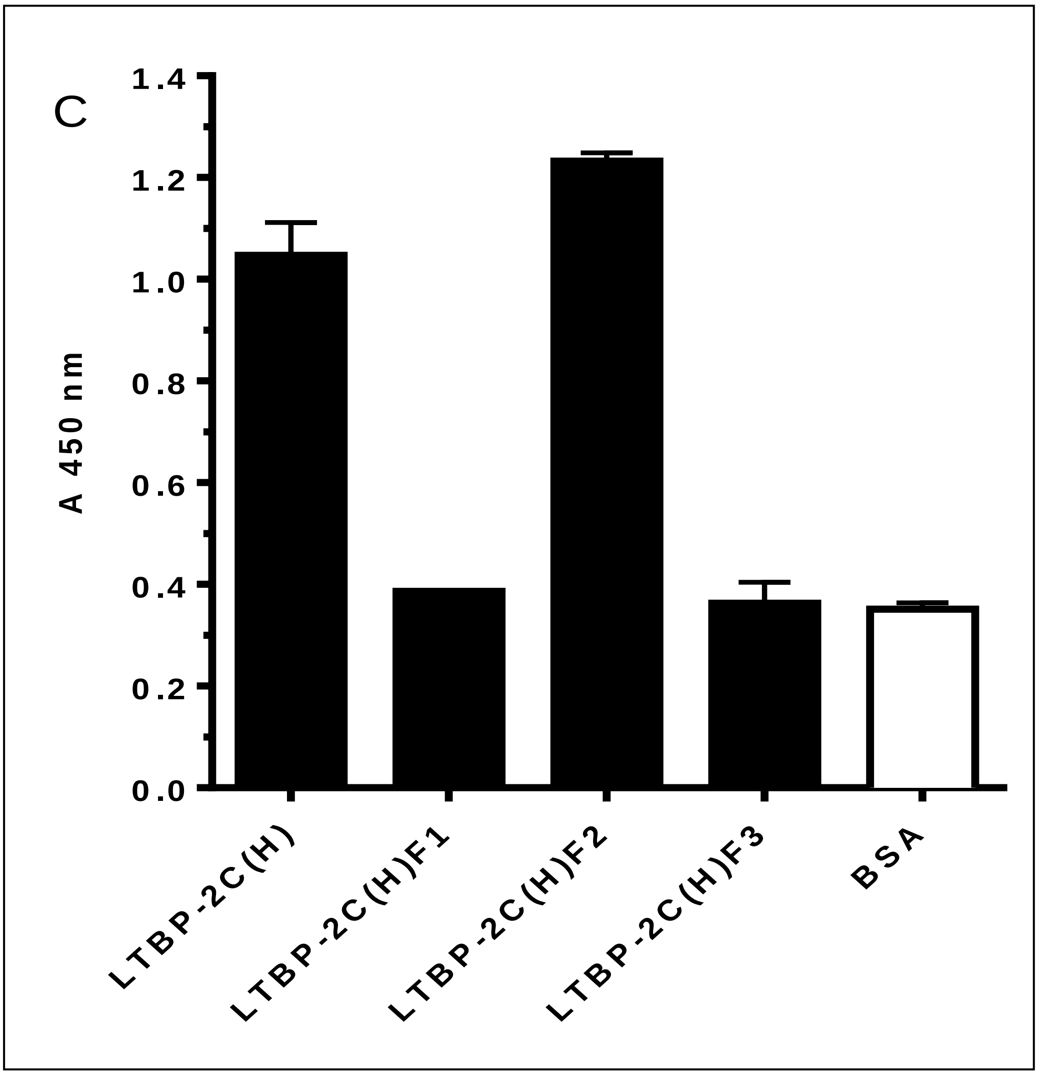

Figure 4. FGF-2 has a single binding domain in the central region of LTBP-2.

C). Three sub-fragments F1, F2 and F3 spanning fragment LTBP-2 C(H) were produced and tested for FGF-2 binding as described under figure 2. LTBP-2C (H) (200 ng/well, 2.4 pmol) or sub-fragment (F1, F2 or F3) (66ng/well, 2.4 pmol) or BSA control was coated on the wells and incubated with FGF-2 (100 ng/ml). Strong specific binding of FGF-2 to sub-fragment LTBP-2C F2 was detected.
