# Supplementary material for: LTBP-2 Has a Single High-Affinity Binding Site for FGF-2 and Blocks FGF-2-Induced Cell Proliferation
Source: PLoS One. 2015 Aug 11;10(8):e0135577. doi: 10.1371/journal.pone.0135577 (PMC4532469; doi:10.1371/journal.pone.0135577)
Supplement: S1 Raw Data — (ZIP) [file pone.0135577.s001.zip › supporting information resubmission 2/Fig 4/Fig 4D.pdf]

bFGF added (ng/ml)	F1			F2			F3			BSA		
0.0	0.049	0.050	0.049	0.056	0.053	0.052	0.051	0.055	0.053	0.052	0.053	0.051
4.0	0.096	0.095	0.104	0.578	0.587	0.512	0.108	0.098	0.113	0.113	0.109	0.110
5.3	0.133	0.130	0.132	0.747	0.647	0.653	0.120	0.122	0.129	0.140	0.145	0.143
7.1	0.144	0.159	0.151	0.850	0.853	0.907	0.175	0.164	0.174	0.177	0.168	0.173
9.5	0.193	0.197	0.212	1.212	1.081	1.076	0.220	0.197	0.229	0.243	0.240	0.242
12.7	0.276	0.263	0.248	1.478	1.316	1.345	0.299	0.232	0.266	0.301	0.290	0.296
16.8	0.394	0.422	0.314	1.830	1.586	1.548	0.355	0.368	0.372	0.388	0.372	0.380
22.5	0.452	0.505	0.517	1.871	1.992	1.864	0.564	0.427	0.471	0.502	0.528	0.515
30.0	0.614	0.669	0.711	2.138	2.226	2.201	0.598	0.608	0.681	0.610	0.614	0.612

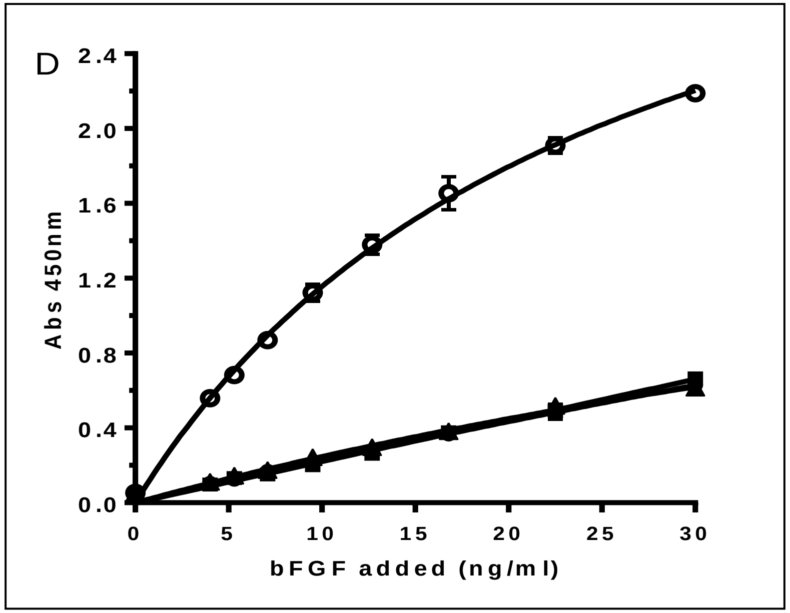

Figure 4. FGF-2 has a single binding domain in the central region of LTBP-2.
D). Subsequently binding curves were obtained for sub-fragments F1 (solid squares), F2 (open circles), F3 (solid circles) (35 ng/well, 1.2 pmol) coated on the wells and incubated with increasing concentrations of FGF-2 (0-30 ng / ml). Note specific FGF-2 binding to sub-fragment LTBP-2C F2 but no binding of fragments F1 and F3 above the BSA control (triangles).
