# Supplementary material for: LTBP-2 Has a Single High-Affinity Binding Site for FGF-2 and Blocks FGF-2-Induced Cell Proliferation
Source: PLoS One. 2015 Aug 11;10(8):e0135577. doi: 10.1371/journal.pone.0135577 (PMC4532469; doi:10.1371/journal.pone.0135577)
Supplement: S1 Raw Data — (ZIP) [file pone.0135577.s001.zip › supporting information resubmission 2/Fig 4/Fig 4E.pdf]

bFGF added (nM)	bFGF bound (fmol)		
0.00	0.00	0.00	0.00
0.23	3.43	3.44	2.95
0.31	4.30	3.61	3.63
0.41	4.84	4.86	5.17
0.55	6.84	5.89	5.87
0.74	8.30	7.12	7.43
0.97	10.10	8.68	8.33
1.31	9.48	10.24	9.48
1.74	10.73	11.32	11.25

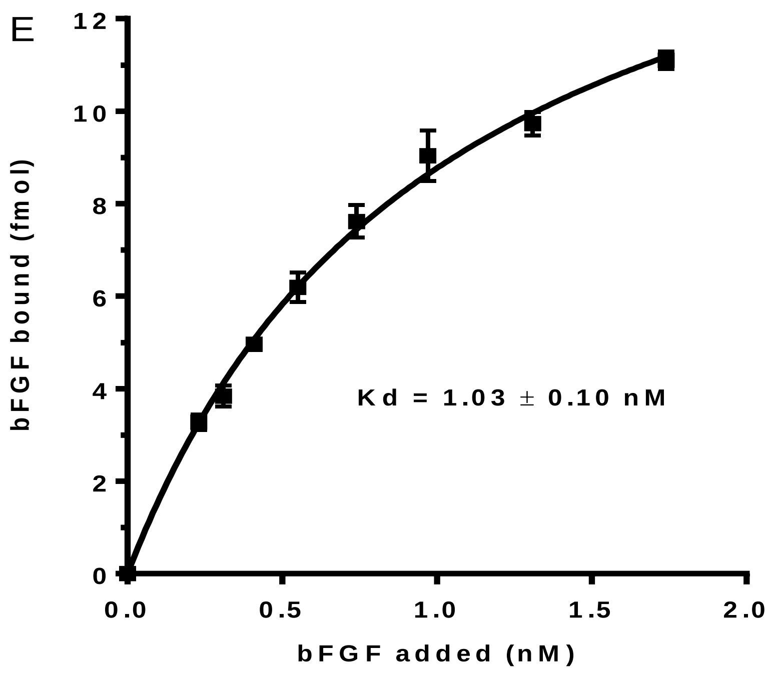

Figure 4. FGF-2 has a single binding domain in the central region of LTBP-2.

E). The Kd for the FGF-2 interaction with sub-fragment LTBP-2C F2 was calculated as 1.03 ± 0.10 nM which is similar to the Kds calculated for the interactions of FGF-2 with full-length LTBP-2 and fragment LTBP2C.
