# Supplementary material for: LTBP-2 Has a Single High-Affinity Binding Site for FGF-2 and Blocks FGF-2-Induced Cell Proliferation
Source: PLoS One. 2015 Aug 11;10(8):e0135577. doi: 10.1371/journal.pone.0135577 (PMC4532469; doi:10.1371/journal.pone.0135577)
Supplement: S1 Raw Data — (ZIP) [file pone.0135577.s001.zip › supporting information resubmission 2/Fig 5/Fig 5.pdf]

LTBP-2C(H)	LTBP-2C(H) F1	LTBP-2C(H) F2	LTBP-2C(H) F3	BSA
1.404	0.258	1.504	0.204	0.176
1.350	0.183	1.481	0.177	0.154
1.308	0.190	1.657	0.235	0.210

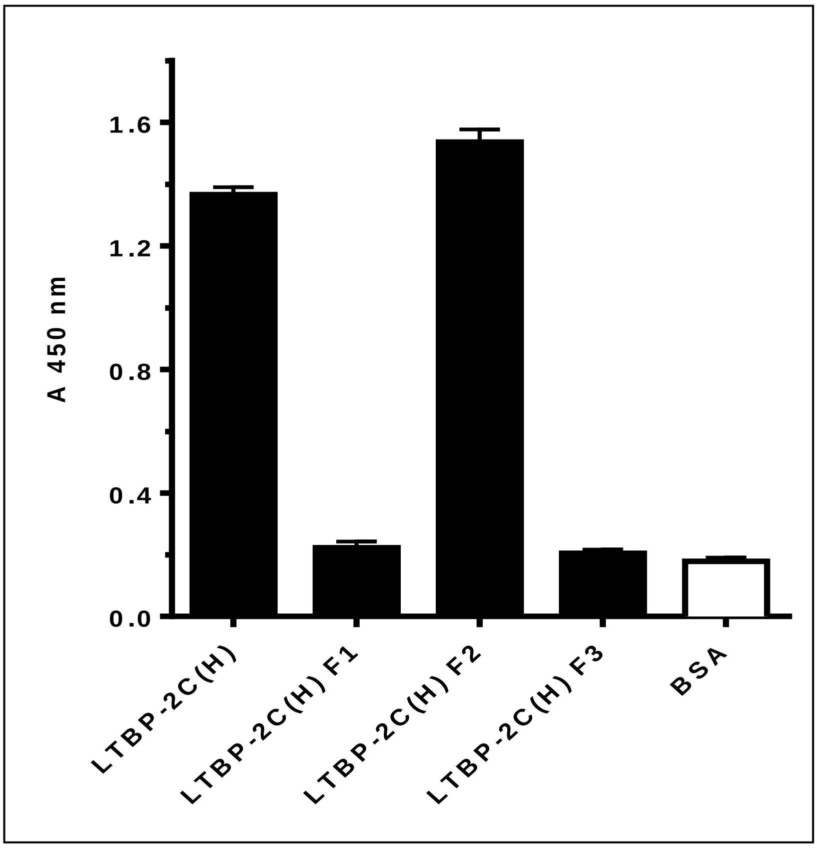

Figure 5. The FGF-2 binding site is close to the central heparin binding site on LTBP-2
In a previous study [32] we identified LTBP-2 C(H) as a heparin-binding fragment of LTBP-2. To further define the location of this heparin binding activity, the three sub-fragments F1, F2, F3 spanning LTBP-2 C(H), were assayed for heparin binding using a heparin-albumin conjugate (HAC). HAC or BSA control (400 ng) was coated on wells followed by incubation with equimolar concentrations (23.5 nM) of LTBP-2C(H) or sub-fragment F1, F2 or F3. Specific binding was detected using anti-His_4 antibody targeting the poly-His tag on each recombinant fragment. Fragment F2 showed strong specific binding to the heparin conjugate in contrast to F1 and F3 which showed no binding above background.
