# Supplementary material for: LTBP-2 Has a Single High-Affinity Binding Site for FGF-2 and Blocks FGF-2-Induced Cell Proliferation
Source: PLoS One. 2015 Aug 11;10(8):e0135577. doi: 10.1371/journal.pone.0135577 (PMC4532469; doi:10.1371/journal.pone.0135577)
Supplement: S1 Raw Data — (ZIP) [file pone.0135577.s001.zip › supporting information resubmission 2/Fig 6/Fig 6A Raw Data.pdf]

| FGF-2 | FGF-2 + Follistatin | FGF-2 + LTBP-2 (5 fold) | FGF-2 + LTBP-2 (10fold) | FGF-2 + LTBP-2C F2 (5fold) |
|---|---|---|---|---|
| 0.971 | 1.106 | 0.682 | 0.571 | 0.901 |
| 1.153 | 1.171 | 0.887 | 0.500 | 0.816 |
| 1.081 | 1.298 | 0.597 | 0.451 | 0.866 |
| 1.134 | 1.113 | 0.691 | 0.489 | 0.906 |
| 1.273 | 1.271 | 0.396 | 0.588 | 0.772 |
| 1.128 | 0.956 | 0.600 | 0.675 | 0.797 |
| 1.286 | 1.136 | 0.589 | 0.539 | 0.805 |
| 0.960 | 1.191 | 0.543 | 0.599 | 0.801 |

| FGF-2 + LTBP-2C F2 (10fold) | Follistatin | LTBP-2 | LTBP-2C F2 | cells only |
|---|---|---|---|---|
| 0.796 | 0.432 | 0.665 | 0.599 | 0.391 |
| 0.729 | 0.347 | 0.691 | 0.575 | 0.458 |
| 0.811 | 0.322 | 0.640 | 0.501 | 0.490 |
| 0.849 | 0.413 | 0.605 | 0.554 | 0.555 |
| 0.822 | 0.507 | 0.596 | 0.526 | 0.529 |
| 0.796 | 0.523 | 0.522 | 0.509 | 0.447 |
| 0.874 | 0.592 | 0.596 | 0.549 | 0.484 |
| 0.862 | 0.558 | 0.547 | 0.475 | 0.433 |

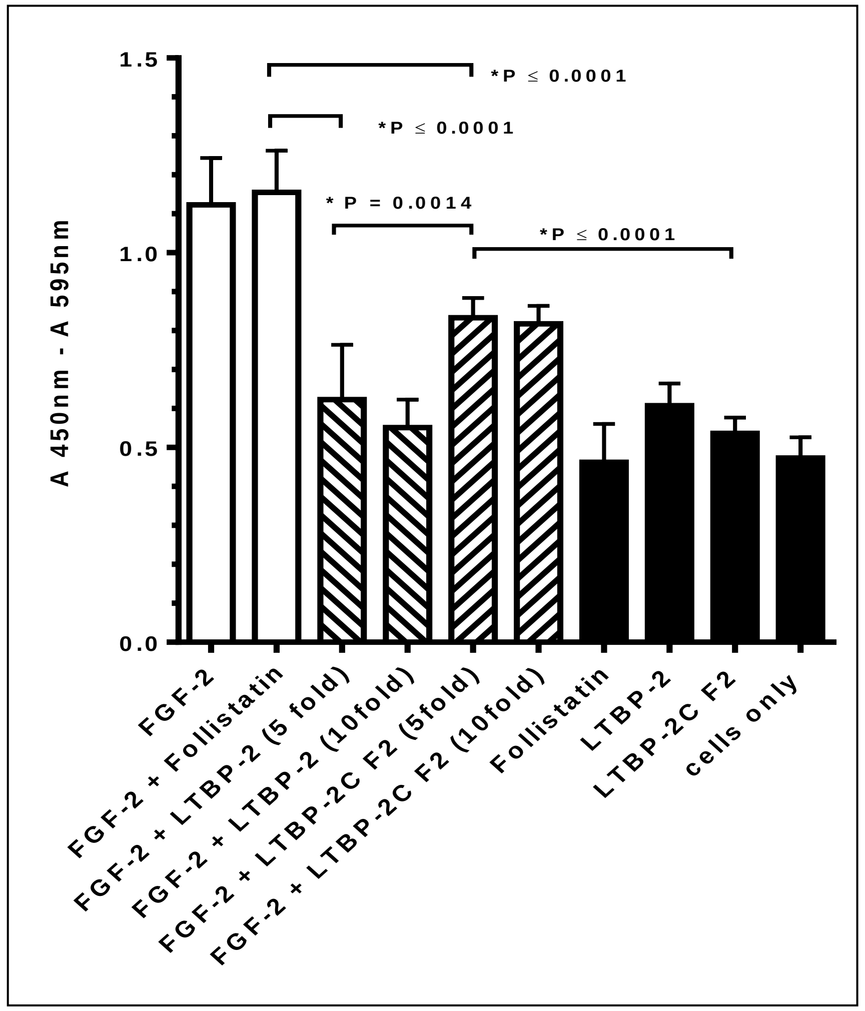


**Figure 6. LTBP-2 blocks FGF-2-induced cell proliferation**

A. The effect of LTBP-2 on the bio-activity of FGF-2 was tested in a cell proliferation assay (see experimental). Human foreskin fibroblasts were treated with FGF-2 with and without follistatin (white columns), or FGF-2 and follistatin pre-incubated with 5 or 10 fold molar excess of full length LTBP-2 or fragment LTBP-2C F2 (cross-hatched). Negative controls (black columns), included cells only and cells incubated with follistatin, LTBP-2 or fragment LTBP-2C F2. Mean values ± S.D. from triplicate determinations. Note 5 fold molar excess of full-length LTBP-2 completely blocked FGF-2 induced cell proliferation (p= 0.0001) and 5-fold molar excess of fragment LTBP-2C F2 partially blocked the activity (p= 0.0001).
