# Supplementary material for: LTBP-2 Has a Single High-Affinity Binding Site for FGF-2 and Blocks FGF-2-Induced Cell Proliferation
Source: PLoS One. 2015 Aug 11;10(8):e0135577. doi: 10.1371/journal.pone.0135577 (PMC4532469; doi:10.1371/journal.pone.0135577)
Supplement: S1 Raw Data — (ZIP) [file pone.0135577.s001.zip › supporting information resubmission 2/Fig 6/Fig 6C Raw Data.pdf]

Signal Intensity	FGF only		FGF + LTBP-2 FL		FGF + LTBP-2C F2		Cells only control	
pFGFR1	18657	19550	3786	3405	7623	7087	3115	3249
Total FGFR1	12450	12664	12756	12776	12806	12882	13404	13455
pFGFR1/total FGFR1	1.4985542	1.5437461	0.2968015	0.2665153	0.5952678	0.5501475	0.2323933	0.2414716
average FGF only	1.521150134							
FGF only adjusted to 100%	100							
percentage relative to " FGF only" signal	98.514559	101.48546	19.511653	17.520648	39.132751	36.166551	15.277475	15.874277

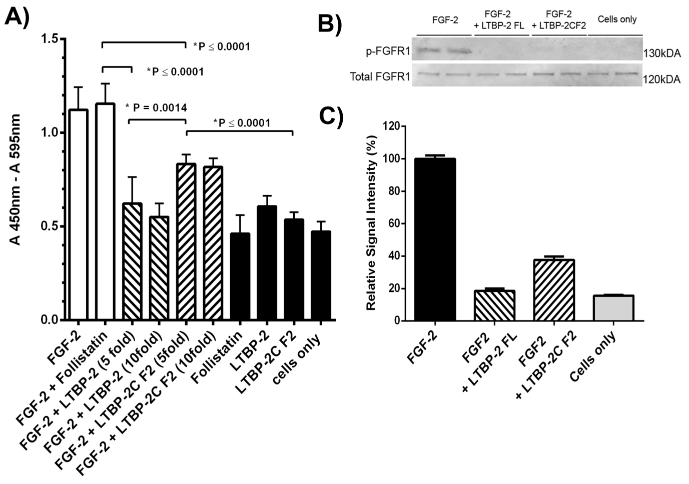

B. Immunoblot analysis FGF receptor (FGFR1) phosphorylation. Human foreskin fibroblasts were treated for 2 hours with FGF-2 (10 ng / ml) only or with FGF-2 plus 10-fold molar excess of full length LTBP-2 (LTBP-2 FL) or fragment F2 (LTBP-2C F2). Control cells had no FGF-2 or LTBP-2 added. Cellular proteins were extracted and duplicate samples were analysed by SDS-PAGE and immunoblotting with anti-phospho-FGFR1 antibody, and anti-total FGFR1 antibody. Bands were visualised using the LI-COR Odyssey Infrared Imaging System.

C. The band intensity was measured using ImageJ 1.48 software [National Institutes of Health (NIH), Bethesda, MD] and normalised to the internal β actin signal. The ratio of the phospho-FGFR1 to total FGFR1 value for each sample is expressed relative to the average FGF-2 only control value (= 100%). Note the strong FGFR1 activation by FGF-2 was substantially blocked by both LTBP-2 C and LTBP-2C F2 fragments. Mean values ± S.D. of duplicate lanes.
