# Supplementary material for: LTBP-2 Has a Single High-Affinity Binding Site for FGF-2 and Blocks FGF-2-Induced Cell Proliferation
Source: PLoS One. 2015 Aug 11;10(8):e0135577. doi: 10.1371/journal.pone.0135577 (PMC4532469; doi:10.1371/journal.pone.0135577)
Supplement: S1 Raw Data — (ZIP) [file pone.0135577.s001.zip › supporting information resubmission 2/Fig 9/Fig 9 Raw Data.pdf]

	Normal Skin											
LTBP-2	1.395	1.593	1.441	1.536	1.538	1.519	1.645	1.734	1.692	1.814	1.837	1.848
FGF-2	2.002	1.734	1.802	1.582	1.584	1.604	1.638	1.658	1.651	1.806	1.793	1.789
Rabbit IgG	0.979	0.977	1.032	0.989	1.015	1.007	1.025	0.999	0.992	1.007	0.997	0.985
Mouseb IgG	0.966	1.030	1.070	0.950	1.010	0.997	1.011	0.950	1.023	1.024	0.997	0.972

	Keloid											
LTBP-2	7.805	7.688	7.673	5.954	5.914	6.798	7.978	8.005	8.123	8.234	7.967	8.117
FGF-2	8.132	7.617	7.619	5.873	6.206	6.810	7.739	7.976	7.574	8.130	7.508	7.705
Rabbit IgG	1.021	1.123	1.314	1.100	1.168	1.063	0.869	0.849	0.900	0.849	0.870	0.876
Mouseb IgG	1.122	1.075	1.149	1.132	1.093	1.141	0.836	0.930	0.865	0.871	0.907	0.879

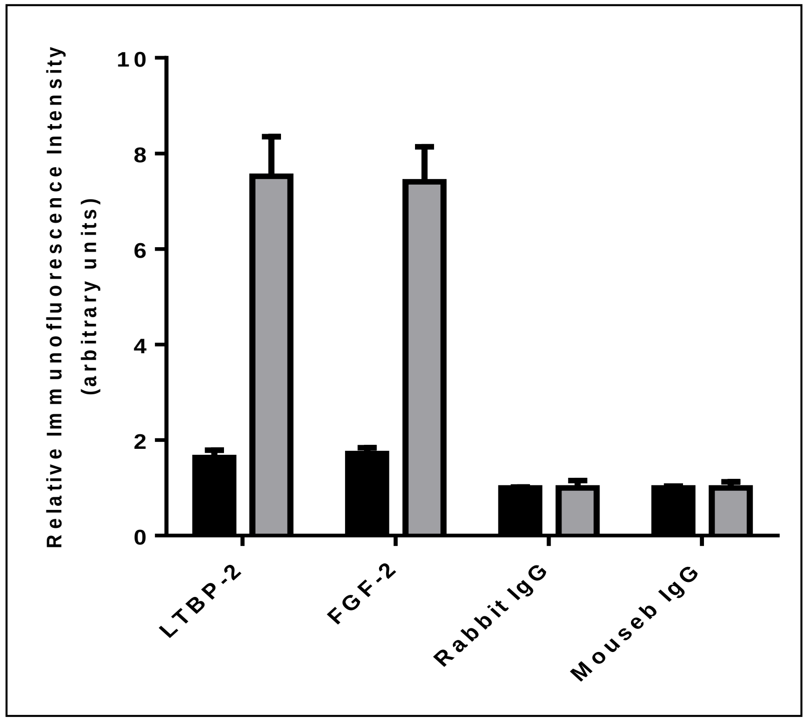

Figure 9. Quantitation of LTBP-2 and FGF-2 in normal skin and keloid.
The relative fluorescence intensities of LTBP-2 and FGF-2 staining (and appropriate IgG controls) in sections of normal human skin (black columns) and keloid (shaded columns) was quantitated from 3 random areas (each 0.038 mm^2) per section using the AnalySIS software package (Soft-Imaging System, Munster, Germany). Values expressed relative to the background control signal (= 1 unit). Mean values ± S.D. of triplicate determination are shown.
